# Strategic complements: Poverty-targeted subsidy programs show additive benefits on household toilet purchases in rural Cambodia when coupled with sanitation marketing

**DOI:** 10.1371/journal.pone.0269980

**Published:** 2022-06-15

**Authors:** Yi Rong Hoo, George Joseph, Rafael Rivera, Susanna Smets, Hanh Nguyen, Per Ljung, Sreymom Um, Georgia Davis, Jeff Albert

**Affiliations:** 1 The World Bank, Washington, DC, United States of America; 2 John F. Kennedy School of Government, Harvard University, Cambridge, MA, United States of America; 3 Thrive Networks / East Meets West, San Francisco, CA, United States of America; 4 Aquaya Institute, San Anselmo, CA, United States of America; University of Maryland College Park, UNITED STATES

## Abstract

While poverty-targeted subsidies have shown promise as a means of reducing financial constraints on low-income populations to invest in new latrines, concerns have been raised about whether they may reduce demand for new latrines among non-eligible, non-poor populations, especially in geographically limited or closed markets. Using quasi experimental methods, we investigate the interaction effects of the “CHOBA” subsidy, a partial poverty-targeted monetary incentive to build a toilet, and a sanitation marketing program (SanMark) on new latrine uptake among households from different income segments in 110 rural villages across six Cambodian provinces. These programs were implemented either jointly with or independently. Overall, we find strong complementarity of the CHOBA subsidy with SanMark where the coupled implementation of the programs increased latrine uptake across all households as compared to exclusive deployment of the programs independently. Additionally, the CHOBA subsidy alone resulted in higher gains among the poor compared to SanMark suggesting that financial constraint is indeed a significant demand barrier for new latrines. The presence of the poverty-targeted subsidies did not reduce demand for new latrine purchases among ineligible households. Instead, we find some evidence for a positive spillover effect of subsidies on uptake of latrines among ineligible households in villages where both programs were implemented indicating that the presence of sanitation subsidies and the decision to purchase latrines among non-beneficiaries can be viewed as complements. We employ multivariate logistic regressions as well as further robustness checks to estimate the effects of the different interventions, with qualitatively consistent results.

## Introduction

In this study, we examine how the co-occurrence of a sanitation marketing intervention (SanMark) and a poverty-targeted subsidies embedded in an integrated behavior change program known as CHOBA, affects outcomes regarding toilet purchase among households living in rural villages in Cambodia. These programs were designed to increase the adoption of proper onsite sanitation facilities among rural households, while moving these households away from practicing open defecation (OD).

As of 2020, nearly 500 million people–primarily in rural areas–practice OD globally. Thus, it is now thought that achieving the Sustainable Development Goal (SDG) of universal access to safely managed sanitation services will require a fifteen-fold increase over the current rates of progress among least developed countries, of which Cambodia is one [1]. In Cambodia, roughly 19% of its population nationally practices OD and to 80% of the most impoverished rural Cambodians practice open defecation [1, 2]. While access to basic sanitation has increased between 2015 and 2020 in the country, Cambodia’s rate of OD nationally is still among the highest in the Southeast Asian region [1].

Reducing open defecation is an important development challenge because open defecation is associated with a whole range of adverse health and socio-economic effects such as diarrheal, helminth and other parasitic infections, dignity, and safety- all of which could have long-lasting implications on a country’s human capital development and productivity [3–13]. The prevalence of open defecation in a community may also negatively impact other households within the same community–even for those who do not practice open defecation [14, 15]. Therefore, it is crucial that interventions are implemented to encourage people to move away from open defecation and increase adoption of proper onsite sanitation options.

Getting people to adopt proper sanitation facilities, such as those facilitating onsite containment of human waste, requires the satisfaction of three criteria: *motivation*, *opportunity*, and *ability* [16]. Consumer *motivation* to install a toilet may be a function of convenience, safety, status, health, custom, or social norms. At the same time, *opportunity* requires having reasonable access to products and services for purchase, and *ability* is principally a function of product affordability and the liquidity households.

In recent years, development efforts to deliver improved sanitation in low-income contexts can be generally divided between demand-side interventions seeking to elevate community-wide *motivation* to end OD and supply-side interventions that offer households the *opportunity* to purchase affordable toilet-related products and services. Perhaps the most widely implemented measure for elevating *motivation* is the Community-Led Total Sanitation (CLTS), an approach intended to stimulate collective action for the eradication of OD that has been executed with the support of large bilateral and multilateral donors in dozens of countries [17].

On the other hand, to increase opportunity for toilet purchase from the supply side, donor-supported efforts to strengthen and nurture private sector product and service providers have focused on Sanitation Marketing (SanMark), which emphasizes the “marketing mix” of “Four Ps”—product, price, place, and promotion [18, 19]. While some of these market-based approaches for sanitation have resulted in hundreds of thousands of sales per intervention, local enterprises may lack sufficient incentives and resources to stimulate demand among households that are less responsive to marketing efforts (*e*.*g*., the poor) [18]. Thus, the potential of SanMark to deliver large numbers of toilets notwithstanding, adoption tends to be considerably lower among the poor, with specific evidence of this pattern in Cambodia [20].

Indeed, neither CLTS nor SanMark explicitly addresses the ability criterion for product adoption. While CLTS has traditionally maintained an anti-consumer subsidy orthodoxy and agnostic towards any toilet product design, SanMark, on the other hand, is focused very specifically on sanitation-related businesses and their engagement of potential customers. However, there has been a growing recognition that liquidity constraints limit the ability to purchase proper sanitation facilities among the rural poor in recent years. Addressing these liquidity constraints is necessary for sustaining sanitation gains in the long run as CLTS may only temporarily reduce OD vis a rapid community-wide effort to install household pit latrines [21].

A straightforward way to increase the ability to purchase a toilet is to reduce its price via a subsidy, and subsidy can take many forms. On one extreme is the direct provision of toilets (or hardware subsidies), including their construction and installation by governments. While such a supply-led approach outright addresses the issue with liquidity constraints, it does not necessarily invoke behavior change or household demand, which in part, has inspired the CLTS approach previously [22, 23]. On the other hand, partial poverty-targeted subsidies are demand-led. Under such a scheme, households are expected to pay the difference between the toilet cost and the subsidy provided. Evidence from Bangladesh as well as India’s Total Sanitation Campaign (TSC) has shown that the demand-driven partial poverty targeted subsidies could result in an increase sanitation uptake among households OD [24, 25]. Similar findings were also reported for Cambodia too previously [26].

As efforts are undertaken to strengthen local sanitation value chains, it is worthwhile to investigate the risks subsidies may pose to demand for latrines in geographically close markets or among those segments of the population where subsidies have not been made available [26]. One hypothetical scenario is that poverty-targeted subsidies can dampen sales of latrines to those not eligible who might instead elect to wait for future financial assistance to make a purchase (in what one might term a market distortion or negative spillover effect).

Still, to our knowledge, evidence in literature so far has suggested otherwise. For example, Nicoletti et al. [26] found that the poverty targeted subsidies had minimal negative impact on non-poor household latrine sales in Cambodia. Moreover, Guiteras et al. [25] detected significant *positive* spillover in Bangladesh, whereby households ineligible for the subsidies increased their toilet adoption as well, with larger increases associated with larger proportion of neighboring households who received subsidy vouchers. This led the authors to believe that toilet subsidies could work as a “social multiplier.” Indeed, the proportion of a community in possession of a private toilet may rival or exceed the importance of a particular household having one with respect to public health. Additionally, several studies have argued for herd protection resulting from collective increases in community-wide toilet coverage [27–30].

Thus, in this study we explore how the co-occurrence of sanitation marketing (SanMark) and poverty-targeted subsidies embedded in an integrated behavior change program affects sanitation coverage in rural villages in six Cambodian provinces. The subsidy program we assess here is known as Community Hygiene Output-Based-Aid (CHOBA), an intervention implemented by the NGO East Meets West/Thrive Networks (EMW) that integrates training of local product/service providers and a partial ($18) poverty-targeted subsidy (offered in Cambodia as an upfront discount to households in possession of a government-issued poverty certificate under the formal “ID-Poor1” and “ID-Poor2” categories) with selected elements of CLTS (specifically, a village meeting intended to elicit disgust with OD). The output basis of the CHOBA subsidy is a rebate to service providers who earn a payment equivalent to the consumer discount upon verification of their installation of a hygienic latrine to each poor household.

On the other hand, the SanMark programs were those executed by the NGOs International Development Enterprises (iDE) and WaterSHED, each of which included novel latrine substructure designs for the Cambodian rural context. The iDE program involves training of latrine business owners as well as financial support of sales agents [26]; the WaterSHED program recruited and trained entrepreneurs as well as employed paid sales agents, while also engaging sub-national and local government officials (including a “civic champions” program) [31].

Our findings suggest that, as expected, the CHOBA subsidy program is more effective at increasing latrine coverage among the poor than SanMark. Also, since it employs no poverty assistance, we find that SanMark, whether executed exclusively or in combination with the CHOBA subsidy program, outperforms the CHOBA subsidy program alone among non-poor households. However, the absence of a decline in performance among the subsidy-ineligible non-poor even with the inclusion of the CHOBA subsidy program is, notable, controverting the hypothesis that the introduction of the CHOBA subsidy program would decrease demand among the non-poor. Instead, we detect no evidence of dampening demand for toilets at market prices resulting from the CHOBA program’s poverty-targeted subsidies. On the other hand, in a finding consistent with those of Guiteras et al. [32], we find evidence to suggest a positive and complementary spillover effect of the CHOBA program on non-poor households. The availability of subsidies for exclusively targeted to low-income households leads to greater purchases of latrines by even relatively higher-income households ineligible for subsidy.

The rest of the study is arranged as follows. We present the methodology of the study in the following section. We discuss the steps involved in constructing our baseline matched cohort of villages using propensity score matching (PSM). Additionally, we also include our methods for the empirical analysis and robustness checks. We then report our findings from the empirical analysis as well as robustness checks in the third section. We present our discussion of the findings in the final section in this study.

## Methods

To examine the interactions between the poverty-targeting CHOBA subsidies and SanMark on new latrine purchase among households, we employ a non-randomized matched cohort design [33, 34] among rural villages in the six Cambodian provinces. The six Cambodian provinces are Kampong Cham, Kampot, Kandal, Kratie, Prey Veng, and Pursat.

We first construct a baseline sample of matched cohort of villages from a universe of villages which received either the CHOBA, SanMark or both programs. We regard each of the program as a treatment. Using a matching approach, we identify statistically similar villages from each of the three treatment groups to construct our eventual matched cohort of villages at baseline. We consider December 2012 as the baseline month of our study. Since we were primarily interested in examining the interaction between the two programs when implemented concurrently in villages, as against them implemented individually, we did not utilize a pure control group of villages that receive none of the programs. Moreover, financial constraints and sample size determined based on power calculations restricted us from adding a pure control group. We then administered a household survey towards the end of the program implementation between August to October 2015 to examine the differences in new latrine purchase among the households over time across different income levels in the matched cohort of villages exposed to the CHOBA subsidy program alone, SanMark alone, or both programs. These households were identified retrospectively in the endline survey to have not owned a latrine prior to December 2012.

**Fig 1:**
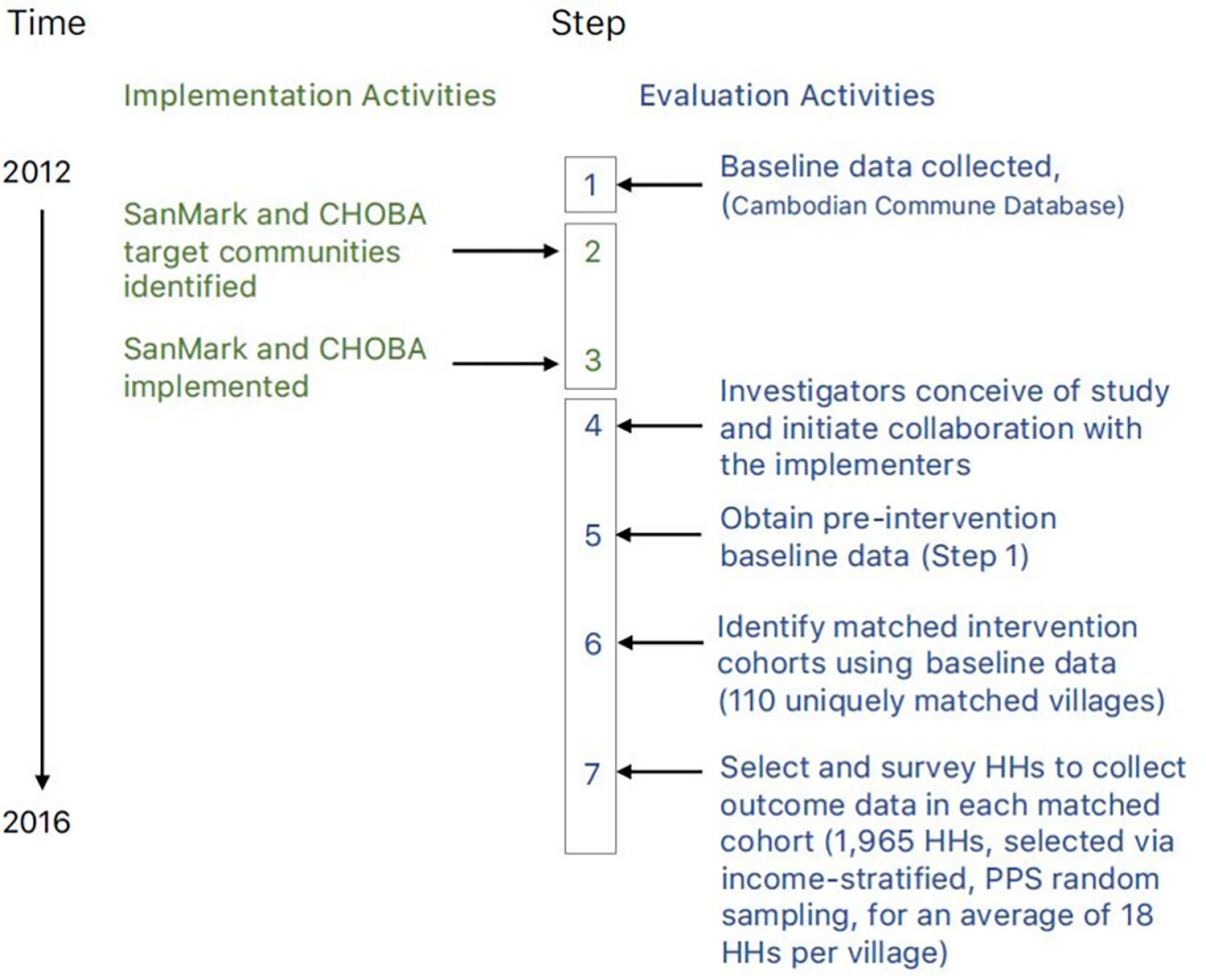
Overview of program implementation & data collection timeline.

The research design is summarized in Fig 1, modeled after the approach laid out by Arnold et al. [35].

### Constructing matched cohorts of villages for baseline

We use the 2012 Cambodia Commune Database (CDB), which is a national database generated by the Cambodian Ministry of Planning and National Committee for Democratic Development to identify villages to be included in the study. The database contains roughly 647 village-level variables on agriculture, demographics, education, employment, commerce, household assets, WASH services status, and health, among others, across all villages in Cambodia. According to the CDB, there are 4,308 villages across the six provinces in which the CHOBA program was implemented. From these villages, we excluded 1,334 villages from consideration that met the following exclusion criteria.

- The occurrence of any previous subsidy or SanMark programs in the village prior to January 2013 since December 2012 was used as the baseline month for our study;
- The offering of large ($50–75) latrine subsidies to the village by the Second Rural Water Supply and Sanitation Sector Project of the Asian Development Bank (ADB).
- Any village exposed to a SanMark program *not* implemented by WaterSHED and iDE, to ensure consistency of SanMark program characteristics and implementation.

Of 2,216 villages that passed this initial screen, we find that the CHOBA program was implemented solely in 497 villages while SanMark was implemented exclusively by Watershed and iDE in another 382 villages. Additionally, 1,337 villages saw the implementation of both programs. All the villages identified here have implemented the programs between January 2013 and 2015, and none before December 2012.

Once the universe of screened villages was identified, we select a subset of villages that are similar in observable characteristics associated with latrine coverage, through a matching process. This matching process is undertaken to avoid the effect of observable confounding variables that might explain any difference in latrine uptake independent of whether the CHOBA program, San Mark, or both interventions were implemented. Thus, in the study sample of villages obtained after matching, we account for nonrandom sampling from a given study population so that the observed covariate distribution is similar across all three treatment groups [35].

Our goal here is to match villages from the three treatment groups with similar characteristics that predict latrine coverage at baseline [36–38]. There are many possible demographic, socioeconomic, and infrastructural characteristics of the villages associated with latrine coverage. The matching approach addresses this challenge by combining covariates into a single “score” or “index” similar to that of a propensity score matching (PSM) approach [33, 34]. We note here that this approach is not strictly a PSM because we are not estimating a propensity score on the treatment assignment but rather a “pseudo-propensity” index estimated on a continuous measure of latrine coverage in each village prior to the interventions instead.

Thus, through a series of multivariate linear regressions (since the outcome is a continuous measure of latrine coverage), we identified 16 variables serving as predictors of village-level latrine coverage at the baseline. These variables are presented in Table 1. Using these variables, we estimate the “pseudo-propensity” score of latrine coverage in each village. Then, the estimated scores are used to identify triads of matched villages from the three groups that have adjacent propensity scores.

**Table 1:** Summary statistics of matched samples.

	(1)	(2)	(3)	(4)	(5)	(6)	(7)	(8)	(9)	(10)	(11)	(12)
		**CHOBA Only**			** **	**SanMark Only**			** **	**Both Programs**		
**Variables**	**Census**		**Matched**		**Census**		**Matched**		**Census**		**Matched**	
**Total Number of Villages**	497		38		382		34		1,337		38	
**Latrine Coverage (%)**	495	0.337	38	0.262	373	0.463	34	0.268	1337	0.323	38	0.253
		(0.275)		(0.160)		(0.307)		(0.165)		(0.262)		(0.159)
**ID-Poor1 & ID-Poor2 Households (%)**	451	0.216	38	0.245	373	0.236	34	0.241	1330	0.276	38	0.245
		(0.132)		(0.060)		(0.118)		(0.055)		(0.126)		(0.058)
**Size of Village (Total Number of families)** ^**+**^	495	287.103	38	291.553	373	211.075	34	180.706	1337	235.750	38	264.947
		(166.825)		(163.769)		(156.416)		(123.913)		(145.321)		(142.973)
**Population older than 46 years old (%)** ^**+**^	495	0.200	38	0.188	373	0.192	34	0.165	1337	0.200	38	0.198
		(0.061)		(0.054)		(0.056)		(0.047)		(0.067)		(0.063)
**Literacy rate (%)** ^**+**^	495	0.034	38	0.030	373	0.034	34	0.027	1337	0.029	38	0.039
		(0.045)		(0.037)		(0.069)		(0.026)		(0.042)		(0.055)
**Rice Farmers (%)** ^**+**^	495	0.183	38	0.188	373	0.154	34	0.173	1337	0.205	38	0.176
		(0.102)		(0.094)		(0.083)		(0.059)		(0.084)		(0.100)
**Population who migrate to work (%)** ^**+**^	495	0.064	38	0.066	373	0.040	34	0.056	1337	0.085	38	0.078
		(0.063)		(0.071)		(0.054)		(0.058)		(0.079)		(0.070)
**Location selling construction materials in commune** ^**+**^	495	0.177	38	0.129	373	0.138	34	0.027	1337	0.137	38	0.210
		(0.641)		(0.593)		(0.421)		(0.156)		(0.519)		(0.490)
**Families without rice land (%)** ^**+**^	495	0.085	38	0.132	373	0.150	34	0.097	1337	0.084	38	0.083
		(0.145)		(0.190)		(0.177)		(0.086)		(0.120)		(0.097)
**Families with motorcycles (%)** ^**+**^	495	0.002	38	0.000	373	0.005	34	0.005	1337	0.004	38	0.003
		(0.007)		(0.001)		(0.012)		(0.012)		(0.010)		(0.007)
**Families with bicycles (%)** ^**+**^	495	0.640	38	0.687	373	0.665	34	0.640	1337	0.681	38	0.692
		(0.303)		(0.330)		(0.295)		(0.216)		(0.271)		(0.285)
**Families with pigs (%)** ^**+**^	495	0.334	38	0.260	373	0.080	34	0.128	1337	0.297	38	0.206
		(0.324)		(0.289)		(0.128)		(0.182)		(0.325)		(0.291)
**Families with cows (%)** ^**+**^	495	0.512	38	0.499	373	0.369	34	0.505	1337	0.434	38	0.380
		(0.351)		(0.347)		(0.303)		(0.276)		(0.291)		(0.331)
**Families with buffaloes (%)** ^**+**^	495	0.043	38	0.077	373	0.044	34	0.126	1337	0.111	38	0.100
		(0.109)		(0.173)		(0.132)		(0.257)		(0.179)		(0.165)
**Families with thatched roof (%)** ^**+**^	495	0.116	38	0.102	373	0.071	34	0.097	1337	0.124	38	0.132
		(0.140)		(0.130)		(0.113)		(0.140)		(0.148)		(0.135)
**Families with piped water (%)** ^**+**^	495	0.301	38	0.433	373	0.391	34	0.497	1337	0.753	38	0.647
		(0.344)		(0.423)		(0.419)		(0.411)		(0.372)		(0.402)
**Women in commune council (%)** ^**+**^	495	0.190	38	0.181	373	0.171	34	0.189	1337	0.155	38	0.131
		(0.094)		(0.096)		(0.098)		(0.096)		(0.091)		(0.075)

Standard deviations in parentheses. Outcome variable is latrine coverage (%).

For the matching process, a 1:1 nearest neighbor matching technique was used. That is, once the pseudo-propensity scores for latrine coverage was calculated for each village, one village each from each of the three treatments with similar or closest scores were added to form the matched triad of villages. The selection of those triads followed a random order matching, which means that the sequence of matching could randomly start with CHOBA, SanMark, or Both-program villages. To find sufficient support across the continuum of estimated scores, we excluded villages with extreme propensity scores on both ends.

**Fig 2:**
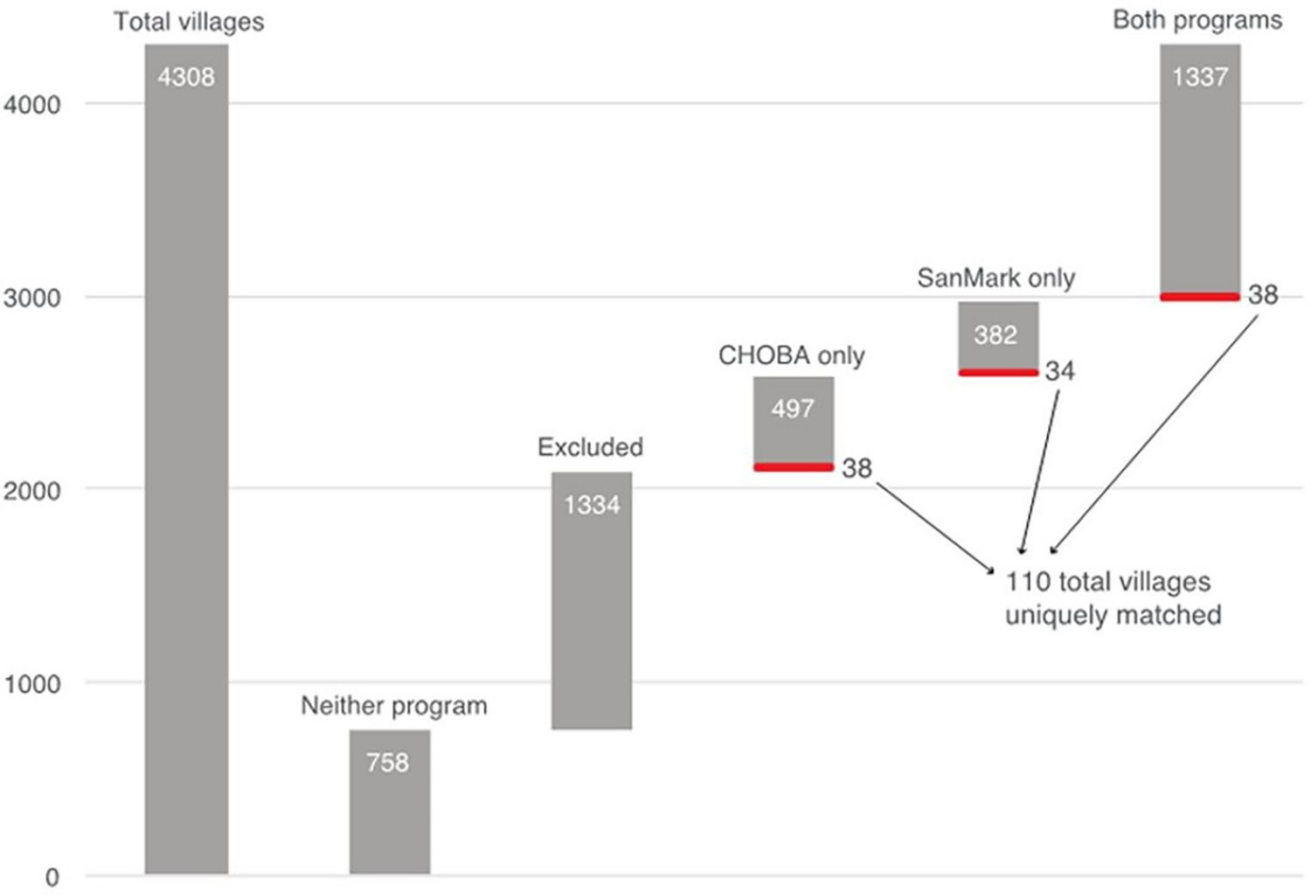
Overview of village screening approach.

Initially, we were able to identify 120 villages from the three groups (40 from each group) that were statistically similar in observed characteristics associated with latrine coverage and poverty levels as of December 2012. However, after closer examination of some of the villages during field visits, we excluded some of them for eventually meeting one of the exclusion criteria mentioned above. Due to logistical constraints, we were not able to replace these villages. Thus, we identify 38 “CHOBA-only” villages, 34 “SanMark-only” villages, and 38 villages where both programs were implemented, respectively, that exhibit similar propensity scores, for a total of 110 uniquely matched villages (Fig 2).

**Fig 3:**
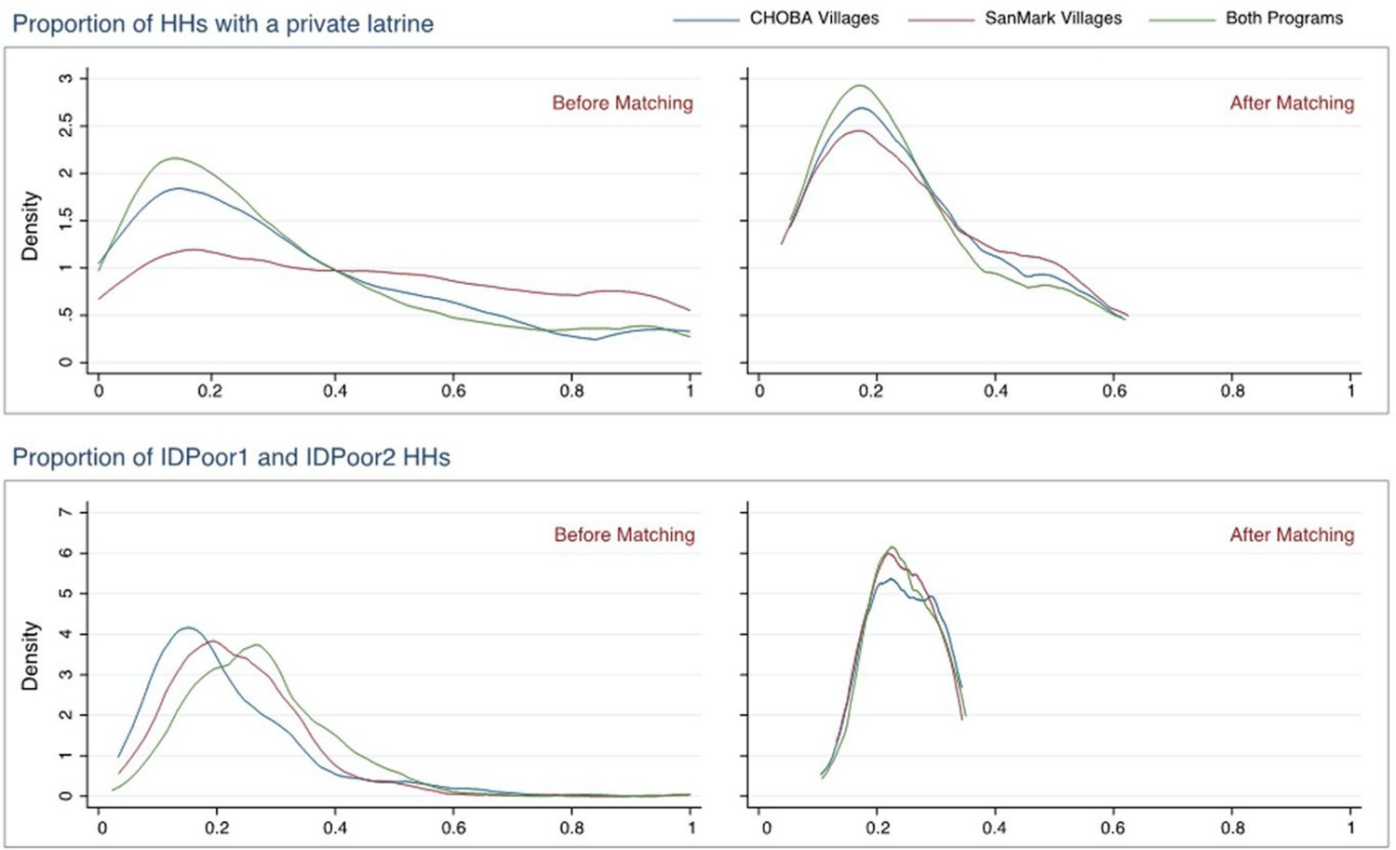
Frequency distributions of latrine coverage and poverty pre- and post-matching.

On average, 26 percent of the households in each of the three intervention groups had already owned a latrine in December 2012 and roughly 24 percent of households were classified as poor households (“IDPoor 1” or “IDPoor 2” according to the Cambodian government’s poverty targeting system) [39]. The frequency distribution for both household characteristics before and after the matching is presented visually in Fig 3. The Identification of Poor Households Program was created in 2006 within the Ministry of Planning in collaboration with the Department of Local Administration (DOLA) of the Ministry of Interior. IDPoor Scores are calculated via a set of socioeconomic indicators of poverty such as household construction materials, asset ownership, and dependency ratio. IDPoor 1 households are considered to be very poor, while IDPoor 2 households are considered moderately poor households. For the purpose of this study however, we will just consider them collectively as poor households. Given that these classifications were determined using socioeconomic indicators rather than income, there may be potential inclusion and exclusion errors. However, any examination of the extent of these errors is beyond the scope of this study.

Although the best matches were selected, differences among the 16 variables between the subgroups remained for some variables. These are presented in Table 2. They are the 1) average number of households per village, 2) average proportion of population above 46 years old, 3) average number of locations selling construction materials, 4) average percentage of families that own a motorcycle, 5) average percentage of families with cows, 6) average proportion of families with piped water, and 7) average proportion of women serving on commune council.

**Table 2:** Comparison of village level characteristics among matched villages at baseline.

	(1)	(2)	(3)	(4)	(5)	(6)	(7)
	CHOBA Only	SanMark Only	Both Programs	T-test			Wald Chi^2^
Variables	Mean/SD	Mean/SD	Mean/Sd	CHOBA vs SanMark	Both vs CHOBA	Both vs SanMark	CHOBA vs SanMark vs Both
Latrine Coverage (%)	0.262	0.268	0.253	-0.006	-0.009	-0.015	0.15
	(0.160)	(0.165)	(0.159)				
ID-Poor1 & ID-Poor2 Households (%)	0.245	0.241	0.245	0.004	0.000	0.004	0.11
	(0.060)	(0.055)	(0.058)				
**Size of Village (Total Number of families)** ^**+**^	291.553	180.706	264.947	110.847***	-26.605	84.241***	12.68***
	(163.769)	(123.913)	(142.973)				
**Population older than 46 years old (%)** ^**+**^	0.188	0.165	0.198	0.023*	0.010	0.033**	7.24**
	(0.054)	(0.047)	(0.063)				
**Literacy rate (%)** ^**+**^	0.030	0.027	0.039	0.003	0.010	0.012	1.55
	(0.037)	(0.026)	(0.055)				
**Rice Farmers (%)** ^**+**^	0.188	0.173	0.176	0.015	-0.012	0.003	0.70
	(0.094)	(0.059)	(0.100)				
**Population who migrate to work (%)** ^**+**^	0.066	0.056	0.078	0.011	0.012	0.022	2.21
	(0.071)	(0.058)	(0.070)				
**Locations selling construction materials in commune** ^**+**^	0.129	0.027	0.210	0.102	0.081	0.183**	5.47*
	(0.593)	(0.156)	(0.490)				
**Families without rice land (%)** ^**+**^	0.132	0.097	0.083	0.035	-0.049	-0.014	2.01
	(0.190)	(0.086)	(0.097)				
**Families with motorcycles (%)** ^**+**^	0.000	0.005	0.003	0.004**	0.003***	-0.001	12.57***
	(0.001)	(0.012)	(0.007)				
**Families with bicycles (%)** ^**+**^	0.687	0.640	0.692	0.047	0.004	0.052	0.96
	(0.330)	(0.216)	(0.285)				
**Families with pigs (%)** ^**+**^	0.260	0.128	0.206	0.131	-0.053	0.078	5.91*
	(0.289)	(0.182)	(0.291)				
**Families with cows (%)** ^**+**^	0.499	0.505	0.380	-0.006	-0.119	-0.125*	3.60
	(0.347)	(0.276)	(0.331)				
**Families with buffaloes (%)** ^**+**^	0.077	0.126	0.100	-0.049	0.023	-0.027	0.95
	(0.173)	(0.257)	(0.165)				
**Families with thatched roof (%)** ^**+**^	0.102	0.097	0.132	0.004	0.031	0.035	1.49
	(0.130)	(0.140)	(0.135)				
**Families with piped water (%)** ^**+**^	0.433	0.497	0.647	-0.064	0.214**	0.150	5.43*
	(0.423)	(0.411)	(0.402)				
**Women in commune council (%)** ^**+**^	0.181	0.189	0.131	-0.008	-0.050**	-0.058***	10.67***
	(0.096)	(0.096)	(0.075)				
Total Number of Villages (N)	38	34	38				

Standard deviations in parentheses. Difference in means is reported for T-tests. Wald Chi^2^ statistic is reported for F-test.

*** p<0.01

** p<0.05

* p<0.1

### Post-implementation outcome measurement

With the matched cohorts of villages from the three subgroups identified, we designed and administered an endline questionnaire to collect post-implementation information on household latrine ownership, sanitation awareness, availability of subsidies and loans, cost and installation processes, and other variables. The questionnaire was implemented in the field between August to October 2015 by an external survey consultant firm with locally based enumerators to conduct the interviews.

The study was approved by the Cambodian National Ethics Committee for Health Research (NECHR). The letter’s approval number was 222 NECHR. The study followed the Standard Operating Procedures (SOP) established by the NECHR. Consent was obtained by enumerators from participating households in the form of oral informed consent. Enumerators were asked to read a pre-approved script for the oral informed consent in the local language which provided the participants the details and background of the study while assuring participants that there were no risks that are associated with participating in the research and that they will not receive any personal benefits. Enumerators were trained to answer any questions and concerns to the participants’ satisfaction. Each informed consent was thus documented on the form signed by the enumerators who were trained to evaluate if the participant voluntarily and knowingly gave informed consent and possessed the legal capacity to give informed consent to participate in this research study. Should participants encounter any issues or concerns, they could contact a member of our research team based in Cambodia.

In total, we recruited 1,965 households, selected by income-stratified, population-proportional-to-size (PPS) random sampling. These households were divided among the 110 villages across Kampong Cham, Kampot, Kandal, Kratie, Prey Veng, and Pursat provinces. On the average about 18 households were sampled per village, with a maximum of 45 households and a minimum of 7 households. Within a given village, the probability of a given household to be selected for participation was proportional to the village population (the number of households per village) and the three income groups. Those income groups are 1) poor (IDPoor 1 and IDPoor 2), 2) “near-poor” (an intermediate poverty category also known as “IDPoor 3”), and 3) non-poor households across the 110 matched villages. To clarify, IDPoor 3 households are those outside of the IDPoor 1 and 2 threshold but still suffering from economic hardship. IDPoor 3 corresponds to roughly 20 percent of the households in each village. A village wide listing was undertaken via a questionnaire designed and executed by the East Meets West (EMW) to identify ID Poor categories based on which the sample of households was drawn to ensure proportional representation from each stratum. In order to account for the complexity of survey design, sample weights were used in the analysis.

The post-implementation outcome survey was powered to detect the following differences in latrine coverage among different income levels across the three cohorts at p = 0.05: 1) Poor: > 6.1 percentage points (pp) 2) Near poor: > 8.6 pp 3) Non-poor: > 6.1 pp and 4) All households: > 3.8 pp.

### Data analysis and empirical framework

We examine several aspects of changes in new latrine ownership within each of the three subgroups and across the three income groups as well (IDPoor 1 and IDPoor 2, Near Poor, and Non-Poor households). For this, we consider three different aspects of new latrine ownership as our outcomes of interest. They are:

1. the likelihood of new latrine purchases among households who did not own a latrine before the start of the program,
2. the likelihood of new latrine purchases and that the latrine was installed and functional at the time of the endline survey, among households who did not own a latrine before the start of the program *(similar to outcome (1) but considering that the latrines were installed and functional)*,
3. the likelihood that the new latrine was installed in a shelter with a durable roof and walls.

Regarding outcome of interest (1), we found that 1,436 of the 1,965 households which were sampled purchased a latrine after January 2013. This could include households who had a latrine previously but have purchased a new one during the project implementation period. Hence, this will be the universe of households for us to examine the effect of the programs on the likelihood that households purchase a new latrine throughout the program implementation. For outcome of interest (2), we defined “installed latrines” as latrines that could be used even if it does not have a shelter or the family has not used it recently. In addition, we asked in the survey if the latrine was functioning at the time of the survey. In other words, installed latrines are available for usage. Overall, only 371 households have newly installed a functioning latrine at the time of the endline survey. Lastly, for outcome of interest (3), We considered walls made of concrete and fibrous cement durable while roofs made of concrete, cement, zinc, and galvanized steel durable. Thus, installed and functioning latrines with both a durable roof and walls are considered durable materials in our definition.

To estimate the effects of the different village programs on the three outcomes of interest described above, we implemented weighted logistic regressions where standard errors are clustered at the village level. To do this, we employ additional covariates comprised of household and village chief characteristics in the empirical model described below. This allows us to further control for potential biases at the household and village chief level not previously accounted for before in our baseline sample construction. Note that we used village level characteristics for the baseline sample construction previously, and we are employing household and village chief characteristics for our empirical analysis below.


$$OUTCOME=\beta_{0}+\beta_{1}\;PROGRAM+\beta_{2}\;INCOME+\beta_{3}\;PROGRAM*INCOME+\beta_{4}\;HHSIZE+\beta_{5}\;HHAWARE+\beta_{6}\;VCAGE+\beta_{7}\;VCFEM+\beta_{8}\;VCEDUC+\beta_{9}VCINVOLVE+\varepsilon$$ (1)

The primary explanatory variable is the different village program (PROGRAM), a categorical variable where the value “1” corresponds to villages with the SanMark program only, “2” to villages with CHOBA only, and “3” to villages where both programs were implemented concurrently. Similarly, the income groups of the household (INCOME) variable are categorical-ordinal, arranged from richest to the poorest where “1” denotes non-poor households, “2” near-poor households (IDPoor3), and “3” poor households (ID-poor 1 & 2).

We include two household characteristics as covariates to account for household level differences potentially affecting latrine purchase, use and quality: household size (HHSIZE) as a continuous measure; and household awareness of the benefits of proper sanitation (HHAWARE) as an index measure computed via principal component analysis (PCA) [40] relying on the following: household exposure to village meeting or a home visit (or both), direct relationship with a latrine seller, knowledge of loan availability for latrine purchase latrine, familiarity with any community-led total sanitation (CLTS) program in the village, and self-reported exposure to latrine and/or handwashing benefits messaging.

Additionally, we include several village chief characteristics as covariates. These covariates were added to account for potential bias arising from village chief characteristics since the implementations of the SanMark and CHOBA programs respectively requires village chief involvement. They are the village chief age (VCAGE), village chief gender (VCFEM), and village chief’s highest education level (VCEDUC). VCAGE is a continuous variable measuring the village chief’s age which ranges between 32 and 77 in our dataset. VCFEM is a dummy variable to indicate female village chiefs. VCEDUC is a dummy variable to indicate if the village chief has a secondary education or higher. We hypothesized that their gender, age, and education could influence our outcomes of interest, in line with literature on leadership styles and the role of village chiefs in CLTS programs [41–43]. Indeed, we find that the village chief education variable was influential in predicting our outcomes of interests. Also, similar to the household awareness measure above, we employ PCA to generate an index measure of village chief involvement throughout the course of the program implementation (VCINVOLVE), based on the following measured variables: village chief visits to households, organization of village meetings, follow-up with latrine orders, participation in fulfilling customer orders for latrines, distribution of communication materials, prioritization of sanitation improvement, prioritization of economic needs of the village, and prioritization of infrastructure investment. For details on the PCA procedure we employed for the HHAWARE and VCINVOLVE variables, see **S1 Text**. The summary statistics for these variables are presented in Table 3.

**Table 3:** Summary statistics at the household level by village programs.

	(1)	(2)	(3)	(4)	(5)	(6)	(7)	(8)	(9)
	SanMark Only			CHOBA Only			Both Programs		
Variables	N	Mean	SD	N	Mean	SD	N	Mean	SD
**Outcomes**									
Household Owns a Latrine (= 1)	656	0.45	0.50	657	0.41	0.49	652	0.55	0.50
Household Purchased a Latrine during Project (= 1)	485	0.25	0.43	469	0.19	0.39	482	0.39	0.49
Latrine is Installed and Working (= 1)	485	0.23	0.42	469	0.18	0.38	482	0.36	0.48
Household’s Latrine has a Durable Wall (Concrete and Cement) (= 1)	109	0.76	0.43	87	0.35	0.48	175	0.53	0.50
Household’s Latrine has a Durable Roof (= 1)	109	0.91	0.29	87	0.67	0.47	175	0.77	0.42
Household’s Latrine has both Durable Wall & Roof (= 1)	109	0.75	0.44	87	0.32	0.47	175	0.52	0.50
**Income Group**									
Non-poor (= 1)	656	0.62	0.48	657	0.56	0.50	652	0.52	0.50
ID Poor 3 (= 1)	656	0.12	0.33	657	0.24	0.43	652	0.23	0.42
ID Poor 1&2 (= 1)	656	0.26	0.44	657	0.21	0.40	652	0.25	0.43
**Household Characteristics**									
Household Size	656	5.39	1.56	657	5.21	1.58	652	5.21	1.70
Household Exposure Index	656	0.25	0.65	657	0.22	0.65	652	0.26	0.61
Household had no exposure (= 1)	656	0.46	0.50	657	0.51	0.50	652	0.45	0.50
Household only attended village meeting (= 1)	656	0.31	0.46	657	0.30	0.46	652	0.25	0.43
Household was visited at house (= 1)	656	0.19	0.39	657	0.18	0.38	652	0.23	0.42
Household attended meeting and was visited (= 1)	656	0.04	0.21	657	0.01	0.10	652	0.07	0.26
Household knows a latrine seller (= 1)	656	0.89	0.31	657	0.90	0.30	652	0.91	0.29
Household knows that loan is available to purchase latrine (= 1)	656	0.95	0.21	657	0.95	0.23	652	0.90	0.30
Household’s village has a CLTS program (= 1)	656	0.79	0.41	657	0.97	0.17	652	0.81	0.39
Household heard of the benefits of latrine (= 1)	656	0.83	0.38	657	0.81	0.40	652	0.84	0.37
Household is aware of the benefits of handwashing (= 1)	656	0.78	0.41	657	0.89	0.32	652	0.95	0.22
**Village Chief Characteristics**									
Village Chief Age	656	0.21	0.41	657	0.23	0.42	652	0.14	0.34
Village Chief is Female (= 1)	656	61.91	7.00	657	58.48	5.25	652	57.18	8.75
Village Chief Education; Secondary School or Higher (= 1)	656	0.09	0.28	657	0.13	0.33	652	0.07	0.25
Village Chief Involvement Index	656	0.94	0.80	657	1.26	0.67	652	1.46	0.73
Village Chief visited households (= 1)	656	0.43	0.50	657	0.62	0.49	652	0.58	0.49
Village Chief organized village meeting (= 1)	656	0.43	0.49	657	0.64	0.48	652	0.62	0.49
Village Chief was asked to follow-up with latrine orders (= 1)	656	0.71	0.45	657	0.75	0.44	652	0.84	0.37
Village Chief helped take orders for latrines (= 1)	656	0.55	0.50	657	0.80	0.40	652	0.84	0.37
Village Chief was asked to distribute communication materials (= 1)	656	0.51	0.50	657	0.46	0.50	652	0.69	0.46
Village Chief prioritizes sanitation in village (= 1)	656	0.50	0.50	657	0.61	0.49	652	0.36	0.48
Village Chief Prioritizes village infrastructure investments in village (= 1)	656	0.53	0.50	657	0.31	0.46	652	0.16	0.37
Village Chief prioritizes economic activities in village (= 1)	656	0.88	0.32	657	0.78	0.42	652	0.96	0.19

Finally, we employ a series of robustness checks for the logistic and linear probability regressions reported above: 1) regression adjustment (RA) [44] 2) inverse propensity weighting (IPW) [45], and 3) inverse propensity weighting with regression adjustment (IPWRA) [46], to confirm the validity of our results. RA models outcomes to account for potential non-random treatment assignment while IPW models treatment to account for non-random treatment assignment. We note here that the propensity scores that are used as weights in the IPW estimations were those that were those from auxiliary regressions at the household level as specified in Model (1). Subsequently, IPWRA models both outcome and treatment to account for non-random treatment assignment (using IPW weights to estimate corrected regression coefficients that are then used to perform regression adjustment). For each of the RA and IPWRA robustness checks, the auxiliary regressions are estimated using both the logistic as well as LPM methods to provide sensitivity checks for the results obtained between those two models.

## Results

In the presentation of results in throughout this section, we follow the recent recommendations against the treating p-values as binary measures of statistical “significance” [29]. Thus, in describing the statistical properties of our findings we present p-values and confidence intervals but also depart from the convention of treating results with p-values above 0.05 as insignificant or null effects. Still, we included the asterisks in our tables simply to indicate results that at the respective p-value thresholds for easy navigation of the results presentation for our readers.

### Estimates of program effects, controlling for household and village chief characteristics

Table 4 presents the predicted probabilities for the three outcomes of interest based on Eq (1) estimated using a logistic regression.: 1) probability of households purchasing a new latrine, 2) probability of households purchasing a new latrine and that the purchased latrine was installed and functional at the time of the endline survey and, 3) among those who purchased a new latrine, the probability that the latrines are constructed with durable materials. We present estimation results that were both not adjusted and adjusted for control variables such as r (INCOME, HHSIZE, HHAWARE, VCAGE, VCFEM, VCEDUC, and VCINVOLVE). These control variables collectively explain roughly 3.3 percent of the total variation for the model estimating new latrine purchases among households. (For complete model results, see Supplemental Materials). In all, the estimated predicted probabilities obtained from the models accounting for the control variables are comparable to the those that were estimated without. For each estimate, we also reported the 95 percent confidence interval in the tables. Robust standard errors are clustered at the village level. Correspondingly, Table 5 presents the estimated marginal effects of SanMark, CHOBA, and the two programs combined, respectively, on the outcomes of interests, controlling for household and village chief characteristics. For the estimated marginal difference, we also present the associated p-values in the brackets. Again, here, we present results that were not adjusted and adjusted for control variables for each estimation.

**Table 4:** Predicted probabilities of outcomes of interest by income groups and village programs.

		(1)	(2)	(3)	(4)	(5)	(6)	(7)	(8)
		Household Purchased Latrine				Latrine was Installed and Working			
		Without Control Variables		With Control Variables		Without Control Variables		With Control Variables	
Sample	Program	Mean/SE	95% CI	Mean/SE	95% CI	Mean/SE	95% CI	Mean/SE	95% CI
**Overall**	SanMark	0.246	(0.183, 0.308)	0.261	(0.207, 0.316)	0.226	(0.167, 0.285)	0.236	(0.184, 0.289)
		(0.032)		(0.028)		(0.030)		(0.027)	
	CHOBA	0.189	(0.137, 0.241)	0.186	(0.138, 0.235)	0.178	(0.132, 0.225)	0.177	(0.130, 0.225)
		(0.026)		(0.025)		(0.024)		(0.024)	
	Both	0.386	(0.308, 0.464)	0.379	(0.297, 0.462)	0.358	(0.283, 0.433)	0.356	(0.276, 0.436)
		(0.040)		(0.042)		(0.038)		(0.041)	
**ID Poor 1&2**	SanMark	0.124	(0.056, 0.192)	0.116	(0.052, 0.180)	0.118	(0.050, 0.186)	0.108	(0.045, 0.171)
		(0.035)		(0.033)		(0.035)		(0.032)	
	CHOBA	0.283	(0.178, 0.388)	0.263	(0.167,0.359)	0.263	(0.159, 0.367)	0.247	(0.149, 0.344)
		(0.054)		(0.049)		(0.053)		(0.050)	
	Both	0.398	(0.295, 0.501)	0.374	(0.272, 0.476)	0.360	(0.263, 0.458)	0.343	(0.246, 0.440)
		(0.053)		(0.052)		(0.050)		(0.049)	
**ID Poor 3**	SanMark	0.382	(0.250, 0.514)	0.412	(0.279, 0.546)	0.367	(0.237, 0.497)	0.389	(0.259, 0.520)
		(0.067)		(0.068)		(0.067)		(0.066)	
	CHOBA	0.313	(0.188, 0.439)	0.322	(0.214, 0.430)	0.292	(0.188, 0.396)	0.301	(0.207, 0.394)
		(0.064)		(0.055)		(0.053)		(0.048)	
	Both	0.428	(0.287, 0.568)	0.439	(0.307, 0.572)	0.395	(0.255, 0.535)	0.408	(0.275, 0.541)
		(0.072)		(0.068)		(0.071)		(0.068)	
**Non-poor**	SanMark	0.273	(0.190, 0.356)	0.277	(0.194, 0.359)	0.246	(0.168, 0.323)	0.243	(0.167, 0.320)
		(0.042)		(0.042)		(0.040)		(0.039)	
	CHOBA	0.094	(0.037, 0.151)	0.098	(0.038, 0.157)	0.092	(0.036, 0.148)	0.097	(0.037, 0.156)
		(0.029)		(0.030)		(0.029)		(0.030)	
	Both	0.360	(0.256, 0.464)	0.359	(0.248, 0.471)	0.339	(0.237, 0.441)	0.342	(0.233, 0.452)
		(0.053)		(0.057)		(0.052)		(0.056)	
**Number of Obs**		1,436		1,436		1,436		1,436	
**Pseudo R-squared**		0.030, 0.062		0.095		0.056		0.089	

		(9)	(10)	(11)	(12)
		**Latrine has Durable Materials**			
		**Without Covariates**		**With Covariates**	
** Sample**	**Program**	**Mean/SE**	**95% CI**	**Mean/SE**	**95% CI**
**Overall**	SanMark	0.746	(0.652, 0.840)	0.621	(0.516, 0.727)
		(0.048)		(0.054)	
	CHOBA	0.324	(0.221, 0.427)	0.389	(0.278, 0.500)
		(0.053)		(0.057)	
	Both	0.517	(0.381, 0.653)	0.543	(0.428, 0.659)
		(0.069)		(0.059)	
**ID Poor 1&2**	SanMark	0.368	(0.119, 0.618)	0.342	(0.083, 0.601)
		(0.127)		(0.132)	
	CHOBA	0.079	(-0.010, 0.168)	0.122	(-0.010, 0.255)
		(0.045)		(0.068)	
	Both	0.399	(0.232, 0.567)	0.482	(0.318, 0.648)
		(0.086)		(0.084)	
**ID Poor 3**	SanMark	0.705	(0.497, 0.913)	0.584	(0.349, 0.819)
		(0.106)		(0.120)	
	CHOBA	0.419	(0.252, 0.587)	0.401	(0.221, 0.581)
		(0.085)		(0.092)	
	Both	0.514	(0.302 0.726)	0.531	(0.364, 0.698)
		(0.108)		(0.085)	
**Non-poor**	SanMark	0.842	(0.757, 0.926)	0.796	(0.693, 0.899)
		(0.043)		(0.052)	
	CHOBA	0.474	(0.257, 0.690)	0.500	(0.268, 0.733)
		(0.111)		(0.119)	
	Both	0.587	(0.425, 0.749)	0.584	(0.442, 0.725)
		(0.083)		(0.072)	
**Number of Obs**		371		371	
**Pseudo R-squared**		0.131		0.186	

Robust clustered standard errors in parentheses. 95% confidence interval is reported beside the estimated means. Sampling weights were applied. For each outcome of interests, refer to specifications (A) in Table 2A in **S1 Table** for full regression results of overall sample without interaction terms & control variables, specifications (B) for full regression results with interaction terms by income group without control variables and specifications (C) for full regression results with interaction term and including other control variables. Control variables included are household size, standardized household awareness index, standardized village chief involvement index, village chief education, village chief age and village chief gender. Estimates were obtained from the respective specifications by income group using the margins postestimation command on STATA.

**Table 5:** Estimated marginal differences of outcomes between village programs by income groups.

		(1)	(2)	(3)	(4)	(5)	(6)	(7)	(8)
		Household Purchased Latrine During Project				Latrine is Installed and Working			
		Without Covariates		With Covariates		Without Covariates		With Covariates	
Sample	Comparisons	Δ /SE/P-value	95% CI	Δ/SE/P-value	95% CI	Δ/SE/P-value	95% CI	Δ/SE/P-value	95% CI
**Overall**	CHOBA vs SanMark	-0.056	(-0.138, 0.025)	-0.075**	(-0.147, -0.002)	-0.047	(-0.122, 0.028)	-0.059*	(-0.129, 0.011)
		(0.041)		(0.037)		(0.038)		(0.036)	
		**[0.173]**		**[0.043]**		**[0.217]**		**[0.096]**	
	Both vs SanMark	0.140***	(0.041, 0.240)	0.118**	(0.011, 0.225)	0.132***	(0.037, 0.228)	0.119**	(0.015, 0.224)
		(0.051)		(0.055)		(0.049)		(0.053)	
		**[0.006]**		**[0.031]**		**[0.006]**		**[0.025]**	
	Both vs CHOBA	0.197***	(0.103, 0.290)	0.193***	(0.097, 0.289)	0.180***	(0.092, 0.268)	0.179***	(0.084, 0.273)
		(0.048)		(0.049)		(0.045)		(0.048)	
		**[0.000]**		**[0.000]**		**[0.000]**		**[0.000]**	
**ID Poor 1&2**	CHOBA vs SanMark	0.159**	(0.034, 0.284)	0.147**	(0.030, 0.263)	0.145**	(0.021, 0.269)	0.139**	(0.023, 0.256)
		(0.064)		(0.059)		(0.063)		(0.059)	
		**[0.013]**		**[0.013]**		**[0.022]**		**[0.019]**	
	Both vs SanMark	0.274***	(0.151, 0.397)	0.258***	(0.135, 0.381)	0.242***	(0.124, 0.3612)	0.235***	(0.118, 0.353)
		(0.063)		(0.063)		(0.061)		(0.060)	
		**[0.000]**		**[0.000]**		**[0.000]**		**[0.000]**	
	Both vs CHOBA	0.115	(-0.032, 0.262)	0.111	(-0.028, 0.225)	0.098	(-0.045, 0.240)	0.096	(-0.041, 0.234)
		(0.075)		(0.071)		(0.073)		(0.070)	
		**[0.125]**		**[0.120]**		**[0.179]**		**[0.169]**	
**ID Poor 3**	CHOBA vs SanMark	-0.069	(-0.251, 0.113)	-0.091	(-0.264, 0.083)	-0.075	(-0.242, 0.092)	-0.089	(-0.250, 0.073)
		(0.093)		(0.088)		(0.085)		(0.083)	
		**[0.458]**		**[0.306]**		**[0.379]**		**[0.028]**	
	Both vs SanMark	0.045	(-0.147, 0.238)	0.027	(-0.168, 0.222)	0.028	(-0.163, 0.219)	0.019	(-0.175, 0.212)
		(0.098)		(0.099)		(0.098)		(0.099)	
		**[0.644]**		**[0.785]**		**[0.773]**		**[0.851]**	
	Both vs CHOBA	0.114	(-0.074, 0.303)	0.118	(-0.053, 0.288)	0.103	(-0.071, 0.277)	0.107	(-0.056, 0.270)
		(0.096)		(0.087)		(0.089)		(0.083)	
		**[0.235]**		**[0.176]**		**[0.247]**		**[0.198]**	
**Non-poor**	CHOBA vs SanMark	-0.179***	(-0.280, -0.078)	-0.179***	(-0.278, -0.080)	-0.154***	(-0.250, -0.058)	-0.147***	(-0.241, -0.053
		(0.051)		(0.051)		(0.049)		(0.048)	
		**[0.001]**		**[0.000]**		**[0.002]**		**[0.002]**	
	Both vs SanMark	0.087	(-0.046, 0.220)	0.083	(-0.063, 0.228)	0.093	(-0.035, 0.222)	0.099	(-0.042, 0.240)
		(0.068)		(0.074)		(0.065)		(0.072)	
		**[0.202]**		**[0.266]**		**[0.154]**		**[0.168]**	
	Both vs CHOBA	0.265***	(0.147, 0.384)	0.261***	(0.134, 0.389)	0.247***	(0.131, 0.364)	0.246***	(0.120, 0.372)
		(0.061)		(0.065)		(0.060)		(0.064)	
		**[0.000]**		**[0.000]**		**[0.000]**		**[0.000]**	
**Number of Obs**		1,436		1,436		1,436		1,436	
**Pseudo R-squared**		0.062		0.095		0.056		0.089	

		**Latrine has Durable Materials**		**Latrine has Durable Materials**	
**Sample**	**Comparisons**	**Without Controls**	** **	**Marginal Diff /SE/P-value**	**95% CI**
**Overall**	CHOBA vs SanMark	-0.422***	(-0.562, -0.283)	-0.233***	(-0.392, -0.073)
		(0.071)		(0.081)	
		**[0.000]**		**[0.004]**	
	Both vs SanMark	-0.229***	(-0.394, -0.063)	-0.078	(-0.243, 0.087)
		(0.084)		(0.084)	
		**[0.007]**		**[0.354]**	
	Both vs CHOBA	0.193**	(0.023, 0.364)	0.155*	(-0.001, 0.311)
		(0.087)		(0.080)	
		**[0.026]**		**[0.052]**	
**ID Poor 1&2**	CHOBA vs SanMark	-0.290**	(-0.555, -0.025)	-0.219	(-0.509, 0.070)
		(0.135)		(0.148)	
		**[0.032]**		**[0.137]**	
	Both vs SanMark	0.031	(-0.279, 0.332)	0.141	(-0.177, 0.459)
		(0.153)		(0.162)	
		**[0.840]**		**[0.385]**	
	Both vs CHOBA	0.321***	(0.131, 0.510)	0.360***	(0.146, 0.574)
		(0.097)		(0.109)	
		**[0.001]**		**[0.001]**	
**ID Poor 3**	CHOBA vs SanMark	-0.285**	(-0.552, -0.018)	-0.183	(-0.469, 0.103)
		(0.136)		(0.146)	
		**[0.036]**		**[0.210]**	
	Both vs SanMark	-0.191	(-0.488, 0.106)	-0.053	(-0.347, 0.241)
		(0.152)		(0.150)	
		**[0.207]**		**[0.724]**	
	Both vs CHOBA	0.094	(-0.176, 0.364)	0.130	(-0.120, 0.380)
		(0.138)		(0.128)	
		**[0.494]**		**[0.308]**	
**Non-poor**	CHOBA vs SanMark	-0.368***	(-0.601, -0.135)	-0.295**	(-0.557, -0.034)
		(0.119)		(0.133)	
		**[0.002]**		**[0.027]**	
	Both vs SanMark	-0.255***	(-0.437, -0.072)	-0.212**	(-0.389,-0.035)
		(0.093)		(0.090)	
		**[0.006]**		**[0.019]**	
	Both vs CHOBA	0.113	(-0.157, 0.384)	0.083	(-0.175, 0.341)
		(0.138)		(0.132)	
		**[0.411]**		**[0.528]**	
**Number of Obs**		371		371	
**Pseudo R-squared**		0.131		0.186	

Robust clustered standard errors in parentheses. P-values reported in brackets 95% confidence interval is reported beside the estimated marginal difference. Sampling weights were applied. For each outcome of interests, refer to specifications (A) in Table 2A in **S1 Table** for full regression results of overall sample without interaction terms & control variables, specifications (B) for full regression results with interaction terms by income group without control variables and specifications (C) for full regression results with interaction term and including other control variables. Estimates were obtained from the respective specifications by income group using the margins postestimation command on STATA.

*** p<0.01

** p<0.05

* p<0.1

Overall, controlling for household and village characteristics, the likelihood for a household to make a new latrine purchase is estimated to be around 26 percent (95% CI: 20.7%, 31.6%) among all households in the SanMark-only subgroup. Among households where CHOBA was implemented exclusively, the likelihood for a new latrine purchase is estimated to be around 19 percent (95% CI: 13.8%, 23.5%). Finally, among households where both programs were jointly implemented, the likelihood of new latrine purchases is estimated to be around 38 percent (95% CI: 29.7%, 46.2%), the highest among the three program interventions. These results are presented in Columns (3) and (4) of Table 4. Also, as shown in the corresponding Columns (3) and (4) of Table 5, the marginal difference (Δ) for the likelihood of new latrine purchases between that of SanMark-only and CHOBA-only subgroups is estimated to be around eight percentage points (pp) (95% CI: 0.2 pp, 14.7 pp; p = 0.043). The estimated effect based on the data we have collected is statistically compatible with the presence of an effect at p<0.05 against the null hypothesis. Additionally, we observe that the likelihood of new latrine purchase when both programs were jointly implemented is superior to that when each program was implemented individually. We find the marginal improvement in the likelihood of new latrine purchase when the programs were implemented together to be 12 pp (95% CI: 1.1 pp, 22.5 pp; p = 0.031) and 19 pp (95% CI: 9.7 pp, 28.9 pp; p<0.001) higher than that of SanMark and CHOBA subgroups respectively. Both estimated marginal differences are statistically compatible with the presence of an effect at p<0.05 and p<0.001, respectively. The greater marginal improvement when both programs were jointly implemented than when each program is implemented individually suggests a synergistic or complementarity between the two approaches. We report similar results when we account for latrine installation and functioning at the time of endline survey in columns (7) and (8) of Tables (4) and (5).

Among poor households (ID poor 1 & 2), we find the likelihood of new latrine purchase to be highest among households when both programs were jointly implemented at 37 percent (95% CI: 27.2%, 47.6%), followed by when CHOBA was implemented exclusively at 26 percent (95% CI: 16.7%, 35.9%) and lastly, among households in the SanMark-only subgroup at 12 percent (95% CI: 5.2%, 18.0%). The marginal difference in likelihood for new latrine purchase among poor households where both programs were jointly implemented as compared to when the SanMark-only subgroup is estimated to be 26 pp (95% CI: 13.5 pp, 38.1 pp; p<0.001). Our data indicates that this difference is statistically compatible at p<0.001. Additionally, we find that the marginal difference in the likelihood of new latrine purchase among poor households in CHOBA-only villages versus that of SanMark-only villages to be 15 pp (95% CI: 3.0 pp, 26.3 pp; p = 0.013), and this is statistically compatible at p<0.05. On the other hand, we also find that implementing both programs together increase the likelihood of new latrine purchase by 11 pp (95% CI: -0.028 pp, 0.225 pp; p = 0.120) but it is not statistically compatible at relevant levels. Taken together, the findings described here suggest that the CHOBA subsidies program works better than the SanMark program at encouraging new latrine purchases among poor households. This also suggest that financial constraint is indeed a significant barrier for latrine purchases among poor households. Furthermore, SanMark does not seem to have an added benefit for poor households when implemented together with the CHOBA program. However, CHOBA program has an incremental, additive effect, primarily due to its effect on the poor, when both programs are implemented concurrently. Again, we find similar results when we consider if the latrines were already installed and functioning at the end of the programs.

Among near-poor households (ID-poor 3) the estimated likelihood of new latrine purchase is highest among households where both programs were jointly at 44 percent (95% CI: 30.7%, 57.2%), followed by that of the CHOBA-only subgroup at 41 percent (95% CI: 27.9%, 54.6%) and finally, that of SanMark-only subgroup at 32 percent (95% CI: 21.4%, 43.0%). The marginal difference in terms of likelihood of new latrine purchases is estimated to be 9.1 pp (95% CI: -26.4 pp, 8.3 pp; p = 0.306) when comparing CHOBA and SanMark; 2.7 pp (95% CI: -16.8 pp, 22.2 pp; p = 0.785) when comparing villages with both programs and SanMark; and 11.8 pp (95% CI: -5.3 pp, 28.8 pp; p = 0.176) when comparing villages with both programs and CHOBA. None of the estimated marginal difference are statistically compatible this time. As discussed earlier, one concern within the WASH-practitioner community here was that the presence of these subsidies could inadvertently dampen or distort the demand for new latrines among subsidy-ineligible households [26]. For example, these households may “wait out” in expectation to receive similar financial benefits in the near future. This is a legitimate concern for program implementers since, these near-poor households could arguably expect that they are “next-in-line” to receive the poverty-targeted subsidies given their socio-economic status. Still, our analysis indicate that this is not a concern. We find that the estimated likelihood of new latrine purchase among near-poor households where both programs were implemented versus that of SanMark-only were similar. This suggests to us that the presence of subsidies have not dampened the demand for new latrines among households who are ineligible. To further add to this discussion, we find that only about seven percent of near-poor households, across the analysis sample (N = 275 of 1,436 households) believed that they will be offered a subsidy in the coming year when we asked them in the survey and an overwhelming majority was not sure if they will be offered the subsidies (see Table A in **S2 Table**). Thus, based on these responses as well as the estimated results above, we believe that, on average, the near-poor households were not “waiting out” in anticipation of receiving a subsidy in the future and the presence of subsidies does not dampen the demand for new latrines among them.

Furthermore, among non-poor households, when both programs were implemented, the likelihood of new latrine purchase is estimated to be 36 percent (95% CI: 24.8%, 47.1%) and 26 pp (95% CI: 13.4 pp, 38.9 pp; p<0.001) higher than that of CHOBA-only subgroup and 8 pp (95% CI: -6.33 pp, 22.8; p = 0.266) higher than that of SanMark-only subgroup. The estimated marginal difference for the former is statistically compatible at p<0.001 while not statistically compatible for the latter. Also, the likelihood of new latrine purchase among non-poor households in SanMark-only villages is around 28 percent (95% CI: 19.4%, 35.9%). This is also roughly 18 pp (95% CI: 0.8 pp, 27.8 pp; p<0.001) higher than that of CHOBA-only villages and is statistically compatible at p<0.001. Again, based on the data collected, we find that the likelihood of new latrine purchase among non-poor households is highest when both programs were jointly implemented. This result, though not surprising, suggests that implementing both programs together in a village could have potential benefits compared to implementing any of the programs individually. Similar to the case for near-poor households discussed above, the findings here also suggest again that the presence of the CHOBA subsidies for poor households did not dampen the demand for new latrines among non-poor households. Finally, taking all the results in this section together, the findings show that the CHOBA subsidies worked better for poor households individually. On the other hand, individually, SanMark, works better for non-poor households.

Lastly, due to sample size constraints, we only discuss the impact of the three programs on latrine durability on the overall sample. We do not attempt to analyze the effect for the various income groups in this section, although we report the estimated figures for each income group. In this regard, we observe that marketing engagements result in more durable latrines among households who purchased new latrines throughout the program implementation. It is estimated that around 62 percent (95% CI: 51.6%, 72.7%) and 54 percent (95% CI: 43.8%, 65.9%) of newly purchased latrines were built with durable materials among households in SanMark-only villages and in villages where both programs were jointly implemented. This compares with only 39 percent (95% CI: 27.8%, 50.0%) of newly purchased latrines among households in CHOBA-only villages.

The marginal difference between that of SanMark villages and CHOBA villages is estimated to be 23 pp (95% CI: 7.3 pp, 23.3 pp; p = 0.004) and is statistically compatible at p<0.01. Additionally, the marginal difference between that of when both programs were jointly implemented and that of CHOBA-only villages is estimated to be around 16 pp (95% CI: -0.1 pp, 31.1pp; p = 0.052). This is statistically compatible at p<0.1. The findings here suggest that marketing engagements could be helpful in promoting households to purchase more durable materials if they are made available.

### Robustness checks

Table 6 presents the estimated average treatment on the treated (ATT) from our robustness checks via the RA, IPW, and IPWRA specifications. We find that our results and subsequent findings t are consistent with those reported in the preceding section. We also report results for the RA and IPWRA model using two sets of auxiliary regressions for further comparison. One of which is estimated using a linear probability model (LPM) as shown in columns (3) and (4) as well as columns (9) and (10), respectively. The other is estimated using a logistic regression model as shown in columns (5) and (6) and columns (11) and (12), respectively. Overall, we find both sets of results to be qualitatively similar. To be consistent with the discussion of results in the preceding section, we will focus on the RA and IPWRA results using estimations from the auxiliary logistic regressions.

**Table 6:** Estimated Average Treatment of the Treated (ATT) of village programs on latrine purchasing and installation by RA, IPW and IPWRA.

		(1)	(2)	(3)	(4)	(5)	(6)	(7)	(8)	(9)	(10)	(11)	(12)
		Household Purchase New Latrine											
Sample	Comparisons	LPM	Logit	RA				IPW		IPWRA			
				(A)		(B)				(A)		(B)	
					95% CI	Δ/SE/P-value	95% CI	Δ/SE/P-value	95% CI	Δ/SE/P-value	95% CI	Δ/SE/P-value	95% CI
**Overall**	CHOBA vs SanMark	-0.067*	-0.075**	-0.065	(-0.148, 0.018)	-0.070	(-0.153, 0.014)	-0.011	(-0.126, 0.104)	-0.048	(-0.126, 0.029)	-0.047	(-0.126, 0.032)
** (N = 1,436)**		(0.037)	(0.037)	(0.042)		(0.043)		(0.059)		(0.040)		(0.041)	
		**[0.077]**	**[0.043]**	**[0.125]**		**[0.103]**		**[0.848]**		**[0.223]**		**[0.246]**	
	Both vs SanMark	0.127**	0.118**	0.143**	(0.030, 0.255)	0.138**	(0.027, 0.248)	0.219***	(0.086, 0.352)	0.170***	(0.069, 0.271)	0.168***	(0.070. 0.267)
		(0.055)	(0.055)	(0.057)		(0.057)		(0.068)		(0.051)		(0.050)	
		**[0.023]**	**[0.031]**	**[0.013]**		**[0.015]**		**[0.001]**		**[0.001]**		**[0.001]**	
	Both vs CHOBA	0.193***	0.193***	0.208***	(0.107, 0.308)	0.207***	(0.108, 0.306)	0.230***	(0.122, 0.338)	0.218***	(0.125, 0.312)	0.215***	(0.122, 0.308)
		(0.049)	(0.049)	(0.051)		(0.050)		(0.055)		(0.048)		(0.047)	
		**[0.000]**	**[0.000]**	**[0.000]**		**[0.000]**		**[0.000]**		**[0.000]**		**[0.000]**	
**ID Poor 1&2**	CHOBA vs SanMark	0.159**	0.147**	0.201***	(0.077, 0.325)	0.223***	(0.096, 0.350)	0.245***	(0.113, 0.378)	0.196***	(0.080, 0.312)	0.198***	(0.058, 0.338)
** (N = 401)**		(0.063)	(0.059)	(0.063)		(0.065)		(0.068)		(0.059)		(0.071)	
		**[0.013]**	**[0.013]**	**[0.001]**		**[0.001]**		**[0.000]**		**[0.001]**		**[0.006]**	
	Both vs SanMark	0.276***	0.258***	0.344***	(0.223, 0.465)	0.366***	(0.244, 0.489)	0.382***	(0.265, 0.499)	0.327***	(0.216, 0.437)	0.331***	(0.184, 0.477)
		(0.067)	(0.063)	(0.062)		(0.063)		(0.060)		(0.056)		(0.075)	
		**[0.000]**	**[0.000]**	**[0.000]**		**[0.000]**		**[0.000]**		**[0.000]**		**[0.000]**	
	Both vs CHOBA	0.117	0.111	0.142**	(0.003, 0.281)	0.143**	(0.005, 0.281)	0.136*	(-0.006, 0.279)	0.131*	(-0.003, 0.265)	0.133*	(-0.001, 0.267)
		(0.074)	(0.071)	(0.071)		(0.070)		(0.073)		(0.068)		(0.068)	
		**[0.117]**	**[0.120]**	**[0.045]**		**[0.042]**		**[0.061]**		**[0.056]**		**[0.051]**	
**Non-poor**	CHOBA vs SanMark	-0.167***	-0.179***	-0.186***	(-0.308, -0.065)	-0.190***	(-0.311, -0.070)	-0.125**	(-0.230, -0.020)	-0.159***	(-0.263, -0.055)	-0.145***	(-0.251, -0.038)
**(N = 760) **		(0.051)	(0.051)	(0.062)		(0.061)		(0.054)		(0.053)		(0.054)	
		**[0.002]**	**[0.000]**	**[0.003]**		**[0.002]**		**[0.020]**		**[0.003]**		**[0.008]**	
	Both vs SanMark	0.091	0.083	0.096	(-0.070, 0.262)	0.089	(-0.073, 0.252)	0.186**	(0.027, 0.347)	0.136*	(-0.011, 0.283)	0.141*	(-0.006, 0.288)
		(0.074)	(0.074)	(0.085)		(0.083)		(0.082)		(0.075)		(0.075)	
		**[0.224]**	**[0.266]**	**[0.258]**		**[0.282]**		**[0.023]**		**[0.070]**		**[0.060]**	
	Both vs CHOBA	0.258***	0.261***	0.282***	(0.145, 0.419)	0.280***	(0.145, 0.414)	0.311***	(0.155, 0.468)	0.295***	(0.163, 0.427)	0.286***	(0.153, 0.418)
		(0.065)	(0.065)	(0.070)		(0.068)		(0.080)		(0.067)		(0.068)	
** **	** **	**[0.000]**	**[0.000]**	**[0.000]**		**[0.000]**		**[0.000]**	** **	**[0.000]**		**[0.000]**	
		**Latrine is Installed and Working**											
**Sample**	**Comparisons**	**LPM**	**Logit**	**RA**				**IPW**		**IPWRA**			
				**(A)**		**(B)**				**(A)**		**(B)**	
** **	** **	** **	** **	**Δ/SE/P-value**	**95% CI**	**Δ/SE/P-value**	**95% CI**	**Δ/SE/P-value**	**95% CI**	**Δ/SE/P-value**	**95% CI**	**Δ/SE/P-value**	**95% CI**
**Overall**	CHOBA vs SanMark	-0.051	-0.059*	-0.052	(-0.128, 0.024)	-0.056	(-0.132, 0.020)	-0.011	(-0.111, 0.090)	-0.041	(-0.011, 0.029)	-0.040	(-0.112, 0.032)
** (N = 1,436)**		(0.036)	(0.036)	(0.039)		(0.039)		(0.051)		(0.036)		(0.037)	
		**[0.160]**	**[0.096]**	**[0.180]**		**[0.149]**		**[0.836]**		**[0.256]**		**[0.276]**	
	Both vs SanMark	0.128**	0.119**	0.143	(0.036, 0.250)	0.137***	(0.033, 0.241)	0.210***	(0.083, 0.338)	0.167***	(0.072, 0.262)	0.161***	(0.070, 0.253)
		(0.054)	(0.053)	(0.055)		(0.053)		(0.065)		(0.049)		(0.047)	
		**[0.020]**	**[0.025]**	**[0.009]**		**[0.010]**		**[0.001]**		**[0.001]**		**[0.001]**	
	Both vs CHOBA	0.178***	0.179***	0.195***	(0.097, 0.293)	0.193***	(0.098. 0.288)	0.221***	(0.111, 0.331)	0.208***	(0.115, 0.300)	0.201***	(0.110, 0.292)
		(0.048)	(0.048)	(0.050)		(0.049)		(0.056)		(0.047)		(0.046)	
		**[0.000]**	**[0.000]**	**[0.000]**		**[0.000]**		**[0.000]**		**[0.000]**		**[0.000]**	
**ID Poor 1&2**	CHOBA vs SanMark	0.153**	0.139**	0.187***	(0.064, 0.311)	0.210***	(0.085, 0.334)	0.227***	(0.097, 0.358)	0.180***	(0.065, 0.295)	0.184***	(0.046, 0.322)
** (N = 401)**		(0.063)	(0.059)	(0.063)		(0.064)		(0.067)		(0.059)		(0.071)	
		**[0.017]**	**[0.019]**	**[0.003]**		**[0.001]**		**[0.001]**		**[0.002]**		**[0.009]**	
	Both vs SanMark	0.254***	0.235***	0.294***	(0.174, 0.413)	0.319***	(0.204, 0.434)	0.341***	(0.228, 0.455)	0.284***	(0.177, 0.392)	0.289***	(0.146, 0.432)
		(0.065)	(0.060)	(0.061)		(0.059)		(0.058)		(0.055)		(0.073)	
		**[0.000]**	**[0.000]**	**[0.000]**		**[0.000]**		**[0.000]**		**[0.000]**		**[0.000]**	
	Both vs CHOBA	0.101	0.096	0.107	(-0.031, 0.245)	0.109	(-0.026, 0.244)	0.114	(-0.031, 0.258)	0.104	(-0.032, 0.239)	0.105	(-0.031, 0.241)
		(0.072)	(0.070)	(0.070)		(0.069)		(0.074)		(0.069)		(0.069)	
		**[0.163]**	**[0.169]**	**[0.129]**		**[0.112]**		**[0.123]**		**[0.134]**		**[0.130]**	
**Non-poor**	CHOBA vs SanMark	-0.135***	-0.147***	-0.151***	(-0.259. -0.042)	-0.154***	(-0.261, -0.047)	-0.107**	(-0.200, -0.015)	-0.127***	(-0.220, -0.034)	-0.114**	(-0.209, -0.019)
**(N = 760) **		(0.049)	(0.048)	(0.055)		(0.055)		(0.047)		(0.048)		(0.048)	
		**[0.007]**	**[0.002]**	**[0.006]**		**[0.005]**		**[0.023]**		**[0.008]**		**[0.018]**	
	Both vs SanMark	0.107	0.099	0.120	(-0.033, 0.274)	0.110	(-0.038, 0.259)	0.195**	(0.041, 0.348)	0.157**	(0.022, 0.292)	0.157**	(0.022, 0.292)
		(0.072)	(0.072)	(0.078)		(0.075)		(0.078)		(0.069)		(0.069)	
		**[0.142]**	**[0.168]**	**[0.124]**		**[0.144]**		**[0.013]**		**[0.022]**		**[0.023]**	
	Both vs CHOBA	0.241***	0.246***	0.271***	(0.136, 0.406)	0.264***	(0.133, 0.395)	0.302***	(0.144, 0.460)	0.284***	(0.152, 0.416)	0.271***	(0.138, 0.404)
		(0.064)	(0.064)	(0.069)		(0.068)		(0.081)		(0.067)		(0.068)	
** **	** **	**[0.000]**	**[0.000]**	**[0.000]**	** **	**[0.000]**	** **	**[0.000]**	** **	**[0.000]**	** **	**[0.000]**	

Robust clustered standard errors in parentheses. P-values reported in brackets. 95% confidence interval is reported beside the estimated marginal difference. Sampling weights were applied. Auxiliary regressions for outcome adjustments of RA and IPWRA in (A) were estimated using linear probability model. Auxiliary regressions for outcome adjustments of RA and IPWRA in (B) were estimated using logistic regressions.

*** p<0.01

** p<0.05

* p<0.1

The robustness checks further reinforce our findings described in the previous section. We find that villages exposed to the CHOBA and SanMark programs implemented together have a higher likelihood to purchase new latrine than those exposed to each program individually. Across all income groups, the increase in the likelihood of new latrine purchase among households in villages exposed to both programs against those in CHOBA-only subgroup is estimated to be around 21 pp (95% CI: 10.8 pp, 30.6 pp; p<0.001) in the RA specification as shown in columns (5) and (6), 23 pp (95% CI: 12.2 pp, 33.8 pp; p<0.001) in the IPW specification as reported in columns (7) and (8) and lastly, 22 pp 23 pp (95% CI: 12.2 pp, 30.8 pp; p<0.001) in the IPWRA estimation reported in columns (11) and (12). Against villages exposed to SanMark, the increase in the likelihood of new latrine purchase is estimated to be 14 pp (95% CI: 2.7 pp, 24.8 pp; p = 0.015) in the RA specification, 22 pp (95% CI: 2.7 pp, 24.8 pp; p = 0.001) in the IPW specification and lastly, 17 pp (95% CI: 7.0 pp, 26.7 pp; p = 0.001) in the IPWRA specification.

The robustness checks also reaffirm our previous findings for both the poor households as well as non-poor households. For poor households, across all three specifications, the CHOBA program is estimated to have increased the likelihood of new latrine purchases by 22 pp (95% CI: 10.8 pp, 30.6 pp; p = 0.006) in the RA specification, 25 pp (95% CI: 11.3 pp, 37.8 pp; p<0.001) in the IPW specification and 20 pp (95% CI: 5.8 pp, 33.8 pp; p = 0.006) compared to the SanMark program. When both programs were implemented, the effect on poor households is estimated to be 37 pp. (95% CI: 24.4pp, 48.9 pp; p<0.001) from the RA specification, 33 pp. (95% CI: 18.4pp, 47.7 pp; p<0.001) from the IPW specification, and 38 pp (95% CI: 26.5 pp, 49.9 pp; p<0.001) from the IPWRA specification.

Among non-poor households, on the other hand, SanMark increased the likelihood of new purchases by 19 pp (95% CI: 7 pp, 31.1 pp; p = 0.002) in the RA specification, 13 pp (95% CI: 2 pp, 23 pp; p = 0.020) in the IPW specification, and 15 pp (95% CI: 3.8 pp, 25.1 pp; p = 0.008) across the three specifications compared to CHOBA. Meanwhile, an additive effect is again evident when the programs are coupled, with the likelihood of latrine purchase among non-poor households is estimated to increase by 28 pp (95% CI: 14.5 pp, 41.4 pp; p<0.001) based on the RA specification, 31 pp (95% CI: 15.5 pp, 46.8 pp; p<0.001) in the IPW specification, and 28.6 pp (95% CI: 15.3 pp, 41.8 pp; p<0.001) in the IPWRA model when compared with that of CHOBA-only subgroup.

Interestingly also, our findings from the robustness checks now suggest that not only the partial poverty-targeted subsidies provided for poor households did not dampen the demand for new latrines among non-poor households, but it also increased latrine demand in what we may term as a “positive spillover” effect, consistent with those of Guiteras et al. [32]. In other words, the presence of the CHOBA subsidies could have encouraged the non-poor households, who were ineligible for the subsidies, to purchase the latrines. Based on the robustness checks, compared with that of the SanMark-only subgroups, households who were exposed to both programs are more likely to purchase new latrines by 19 pp (95% CI: 2.7 pp, 34.7 pp; p = 0.023) based on the IPW specification, and 14 pp (95% CI: 0.6pp to 28.8 pp; p = 0.060). We do note however, that the estimated magnitude of the effect from the RA model is comparatively lower at around 9 pp (95% CI: -7.4 pp, 25.2 pp; p = 0.282), though the directions are similar.

For the complete results of the auxiliary regressions used for the robustness checks, refer to Tables 3A to 3E in S1 Table. In short, we consistently find robust results indicating incremental benefits in encouraging household demand for latrine when both the SanMark and CHOBA programs are introduced together in the villages compared to when either program is implemented individually. Also, our findings consistently show that the SanMark program works relatively better for non-poor households while the CHOBA subsidy program works rather better for poor households.

## Discussion and conclusion

As our findings suggest, the CHOBA subsidy program is more effective at encouraging new latrine purchases among the poor than SanMark. Meanwhile, insofar as it employs no poverty assistance, it is unsurprising that SanMark, whether executed exclusively or in combination with the CHOBA subsidy program, outperforms the CHOBA subsidy program alone among non-poor households. Additionally, the absence of a decline in performance among the subsidy-ineligible households (near-poor & non-poor households) even with the inclusion of the CHOBA subsidy program is, notable, controverting the hypothesis that introducing the CHOBA subsidy program would distort the demand for latrines among the subsidy-ineligible households. Based on the data we analyzed, we detect no evidence of dampening demand for toilets at market prices resulting from the CHOBA program’s poverty-targeted subsidies. Our findings are also similar with that of another experiment from rural Cambodia [26], which found comparable increases in latrine uptake when adding a poverty-targeted subsidy to an existing SanMark program, without associated losses in purchases by non-poor households.

Additionally, in a finding consistent with those of Guiteras et al. [32], there is some evidence, especially from the robustness checks, that suggests that the presence of poverty-targeting subsidies has a positive and complementary spillover effect. the availability of subsidies for low-income households may have also led to increased purchases of latrines by the relatively higher-income households ineligible for subsidy. A potential explanation for this is that while subsidies directly reduce price constraints and thus increase demand among targeted beneficiaries, they also indirectly affect the purchasing decision of non-beneficiaries. Holding price constant, a household may become more likely to invest in a new latrine if a larger fraction of its community is also offered subsidies. Thus, subsidies to encourage latrine adoption and adoption decisions of others can be strategic complements.

Other data collected in our 2015 household survey (and information gleaned from interviews with chiefs of the 109 matched villages) strongly suggest that cost remains a significant constraint on latrine purchase in rural Cambodia. Households without latrines at the time of our survey reported overwhelmingly that either a cost reduction or the offer of a subsidy would most likely inspire a purchase. These findings were also reinforced by village chiefs across all three program interventions. On the other hand, when the privately-driven supply-side opportunity is available through the marketing of sanitation products (SanMark), we find that more of the newly purchased latrines were installed with more durable materials. This potentially demonstrates the effectiveness of supply-side incentives in promoting the use of better-quality materials.

Based on these results, the coupling of carefully designed sanitation subsidies with sanitation marketing programs offers a significant opportunity for additive benefits. SanMark has proven effective (and highly cost-effective) at delivering large numbers of latrines, but it has faced obstacles in the penetration of low-income segments. Even partial subsidies targeted to economically disadvantaged populations should be considered complementary to SanMark programs and as an intervention with strong potential to remove the affordability and liquidity constraints faced by the rural poor. This is especially important in the context of integrated programming that includes demand creation via community engagement and disbursed on an output basis to maximize accountability.

There are several limitations to our study. First, given the circumstances surrounding the project implementation, we were unable to include a pure control or comparison group of villages that does not receive any of the programs examined in this study. So, we were unable to examine if SanMark or CHOBA is better at encouraging latrine purchases than the no program alternative in our study. However, based on the evidence from our study, we can suggest that implementing any of these interventions will have positive effect on latrine purchases rather than not implementing any at all. Further, each program has its comparative advantage in encouraging new latrine uptake for specific population groups.

Secondly, we note that we did not examine the role or include remittances and microfinance loans in our empirical analyses. We acknowledge that these variables could be important given that the availability of remittances and microfinance could alleviate the financial constraints in encouraging latrine uptake among rural households. Some studies in the past have shown empirical evidence to suggest the that the availability of remittances and loans could influence the pattern of household expenditure and increase the willingness to pay for non-food durable goods such as toilets among households [47–52]. Our survey was not designed to accurately measure the availability of loans for households as it was reported based on the household’s knowledge. Nevertheless, when we ran the models to include the availability of micro-credit loans as reported by the households as an additional covariate, we find basically no change to our results. These results can be made available upon requests.

Lastly, while our study has indicated that the presence of the poverty-targeted subsidies did not dampen the demand of new latrine purchase among ineligible households, we suspect that there may be a temporal aspect to the demand distortion. One hypothesis here is that perhaps in the nearer term, subsidy-ineligible households may be more inclined to employ a “wait-and-see” approach in anticipation of potentially receiving a subsidy in the nearer term as compared to the longer term when they may begin to realize that the subsidies will not be given to them after all. In our study, we have only implemented one round of household survey towards the end of the program lifespan which is roughly three years from our designated baseline. Thus, we may have simply measured a “die-off” effect of the market distortion where households no longer wait out for the subsidies and demand has normalized. Perhaps, administering surveys between the start and end of the program could allow this examination. This may have several programmatic implications. Program implementers may need to consider the duration by which say the poverty-targeted subsidies are offered to eligible households. Following the temporal hypothesis, the program may need to be offered long enough until the demand for new latrine among ineligible households normalizes. Additionally, we expect the hypothesized temporal aspects of market distortion effect as a result of the subsidies may be different in communities that are more homogenous socio-economically versus more heterogeneous communities. Perhaps, these are the questions that merit further examination and discussion.

## Supporting information

S1 Text Principal component analysis for village chief involvement and household awareness. (PDF)

S1 Table Full regression tables. (PDF)

S2 Table Household responses when they were asked if they think that they will be offered a subsidy by another organization in the future. (PDF)
