# Supplementary material for: Strategic complements: Poverty-targeted subsidy programs show additive benefits on household toilet purchases in rural Cambodia when coupled with sanitation marketing
Source: PLoS One. 2022 Jun 15;17(6):e0269980. doi: 10.1371/journal.pone.0269980 (PMC9200298; doi:10.1371/journal.pone.0269980)
Supplement: S1 Text — (PDF) [file pone.0269980.s001.pdf]

S1 Text. Principal Component Analysis for Village Chief Involvement and Household Awareness.

Table A: Variable Loadings for Village Chief Involvement and Household Awareness Indices via PCA using Covariance Matrix

**Village Chief Involvement Index**	**Loadings**
Village Chief visited households (=1)	0.494
Village Chief organized village meeting (=1)	0.486
Village Chief was asked to follow-up with latrine orders (=1)	0.341
Village Chief helped take orders for latrines (=1)	0.374
Village Chief was asked to distribute communication materials (=1)	0.400
Village Chief prioritizes sanitation in village (=1)	-0.141
Village Chief prioritizes infrastructure investments in village (=1)	-0.272
Village Chief prioritizes economic activities in village (=1)	0.098
**Covariance Matrix Eigenvalue: 0.583**	
**Household Awareness Index**	**Loadings**
Household had no exposure (=1)	-0.741
Household only attended village meeting (=1)	0.534
Household was visited at house (=1)	0.183
Household attended meeting and was visited (=1)	0.024
Household knows a latrine seller (=1)	0.070
Household knows that loan is available to purchase latrine (=1)	0.059
Household's village has a CLTS program (=1)	0.064
Household heard of the benefits of latrine (=1)	0.327
Household is aware of the benefits of handwashing (=1)	0.116
**Covariance Matrix Eigenvalue: 0.409**	

As part of our analysis, we included proximate measures of village chief involvement and household awareness. These measures were indices constructed using principal component analysis (PCA) (Filmer & Pritchett, 2001). The PCA method allows us to summarize the correlations among a set of observed variables into a single linear combination or index.

Using the PCA method, the village chief involvement index was derived from 8 separate dummy variables that measures different areas of involvement by the village chief. The variables were indicators for 1) village chief visited the household 2) village chief organized a village meeting 3) village chief was asked to follow-up with latrine orders 4) village chief helped take orders for the latrines 5) village chief was asked to distribute communication materials as well as a series of dummy variables that indicated the village chiefs priorities including 6) sanitation in village 7) infrastructure investments in the village and 8) economic activities in the village.

Additionally, we attempted to measure how much exposure to information relating to the different sanitation programs each household had experienced throughout the program. For this, we derived a household exposure index using principal component analysis again from 9 separate dummy variables. They are indicators for

households 1) who had no exposure 2) only attended village meeting 3) visited at house 4) attended a meeting and was visited 5) knows a latrine seller 6) knows that loan is available to purchase latrine 7) village has a CLTS program 8) heard of benefits of latrine and 9) aware of the benefits of handwashing.

Table A summarizes the variables' loadings for both indices. Fig A presents the kernel distribution of both indices. PCA can be based on either the covariance or the correlation matrix. Since all the variables that made up each index respectively were on the same scale, we used the covariance matrix instead (Jolliffe & Cadima, 2016). Each index accounted the largest fraction of the total variances of its respective variables. The inclusion of the indices in our regression analyses was not intended to provide any sort of significant inference but to account for the potential relationship with our outcome of interests in our regression analyses as additional covariates to reduce the bias (if any) of our treatment indicators. Lastly, we standardized both indices prior to including them into our regression analyses.

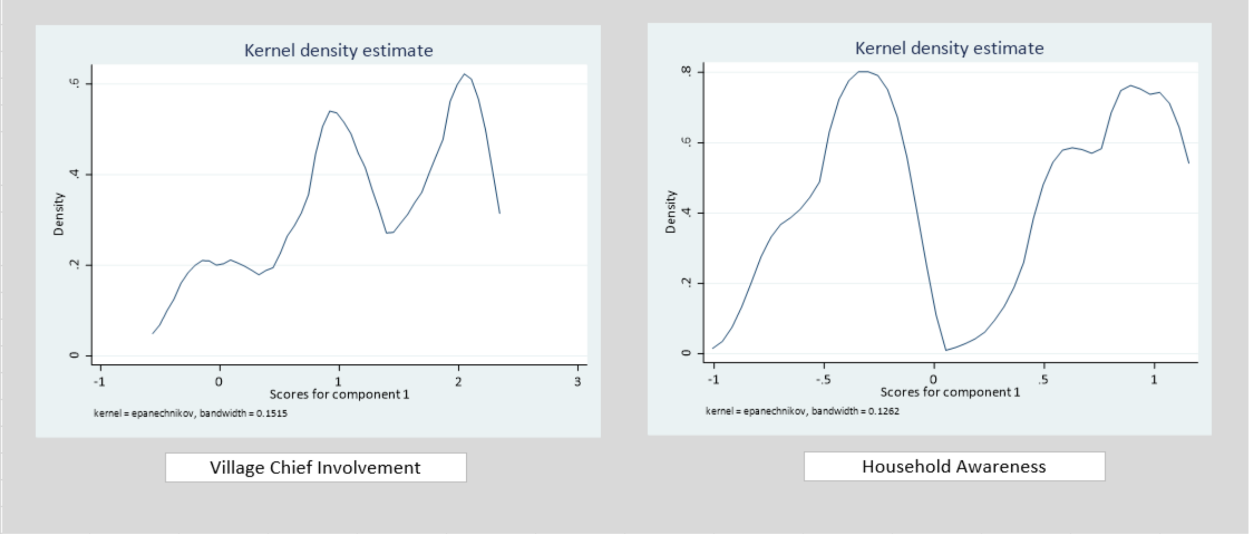

<u>Figure A: Kernel Densities of Village Chief Involvement and Household Awareness Indices</u>

<u>References</u>

Filmer, D., & Pritchett, L. H. (2001). Estimating wealth effects without expenditure data—or tears: an application to educational enrollments in states of India. *Demography*, *38*(1), 115-132.

Jolliffe, I. T., & Cadima, J. (2016). Principal component analysis: a review and recent developments. *Philosophical Transactions of the Royal Society A: Mathematical, Physical and Engineering Sciences*, *374*(2065), 20150202.
