# Supplementary material for: Strategic complements: Poverty-targeted subsidy programs show additive benefits on household toilet purchases in rural Cambodia when coupled with sanitation marketing
Source: PLoS One. 2022 Jun 15;17(6):e0269980. doi: 10.1371/journal.pone.0269980 (PMC9200298; doi:10.1371/journal.pone.0269980)
Supplement: S1 Table — (PDF) [file pone.0269980.s002.pdf]

S1 Table. Full Regression Tables

Table 2A. Logistic Regression Specifications for Results Reported in Tables 4 & 5

	(1)	(2)	(3)	(4)	(5)	(6)	(7)	(8)	(9)
	**Household Purchased Latrine**			**Latrine is Installed and Working**			**Latrine has Durable Materials**		
**Specifications:**	(A)	(B)	(C)	(A)	(B)	(C)	(A)	(B)	(C)
**<u>Village Treatment</u>**									
**(SanMark = 0)**									
Choba Only (=1)	0.717	0.277***	0.272***	0.745	0.310***	0.322***	0.163***	0.169***	0.227**
	(0.175)	(0.112)	(0.110)	(0.176)	(0.126)	(0.131)	(0.057)	(0.093)	(0.144)
	**[0.171]**	**[0.001]**	**[0.001]**	**[0.213]**	**[0.004]**	**[0.005]**	**[0.000]**	**[0.001]**	**[0.020]**
Both (=1)	1.931***	1.497	1.494	1.914***	1.575	1.656	0.365***	0.268***	0.329**
	(0.464)	(0.471)	(0.534)	(0.458)	(0.498)	(0.599)	(0.137)	(0.126)	(0.157)
	**[0.006]**	**[0.200]**	**[0.262]**	**[0.007]**	**[0.151]**	**[0.163]**	**[0.007]**	**[0.005]**	**[0.020]**
**<u>Income Groups</u>**									
**(Non-Poor = 0)**									
Near Poor/ID-Poor 3 (=1)		1.647	1.891*		1.780*	2.048**		0.449*	0.330**
		(0.589)	(0.698)		(0.615)	(0.724)		(0.202)	(0.167)
		**[0.163]**	**[0.084]**		**[0.095]**	**[0.043]**		**[0.076]**	**[0.029]**
Poor/ID-Poor 1&2 (=1)		0.377***	0.332***		0.411**	0.364**		0.110***	0.109***
		(0.127)	(0.121)		(0.151)	(0.144)		(0.075)	(0.089)
		**[0.004]**	**[0.003]**		**[0.016]**	**[0.011]**		**[0.001]**	**[0.006]**
**<u>Interaction Terms</u>**									
Choba*Near Poor (=1)		2.658	2.433		2.294	2.054		1.787	1.939
		(1.608)	(1.457)		(1.327)	(1.178)		(1.373)	(1.779)
		**[0.106]**	**[0.138]**		**[0.151]**	**[0.209]**		**[0.450]**	**[0.470]**
Choba*Poor (=1)		10.034***	10.301***		8.586***	8.691***		0.866	1.065
		(5.203)	(5.489)		(4.690)	(4.861)		(0.867)	(1.220)
		**[0.000]**	**[0.000]**		**[0.000]**	**[0.000]**		**[0.886]**	**[0.956]**
Both*Near Poor (=1)		0.807	0.752		0.715	0.655		1.653	2.396
		(0.410)	(0.388)		(0.362)	(0.333)		(1.036)	(1.586)
		**[0.673]**	**[0.581]**		**[0.509]**	**[0.406]**		**[0.422]**	**[0.187]**
Both*Poor (=1)		3.118***	3.221**		2.674**	2.753**		4.259*	5.812*
		(1.371)	(1.512)		(1.239)	(1.351)		(3.326)	(5.231)
		**[0.010]**	**[0.013]**		**[0.034]**	**[0.039]**		**[0.063]**	**[0.051]**
Household Size			1.110***			1.135***			0.996
			(0.042)			(0.043)			(0.085)
			**[0.005]**			**[0.001]**			**[0.963]**
HH Awareness Index			1.452***			1.404***			0.538***
(Standardized)			(0.117)			(0.110)			(0.075)
			**[0.000]**			**[0.000]**			**[0.000]**
Village Chief Involvement									
Index			1.000			0.974			0.964
(Standardized)			(0.115)			(0.114)			(0.167)
			**[0.998]**			**[0.824]**			**[0.834]**
Village Chief Secondary									
Education or higher (=1)			0.619*			0.586**			1.369
			(0.161)			(0.155)			(0.459)
			**[0.065]**			**[0.043]**			**[0.349]**
Village Chief Age			1.006			1.012			1.054**
			(0.015)			(0.015)			(0.023)
			**[0.663]**			**[0.417]**			**[0.016]**
Female Village Chief (=1)			1.345			1.265			1.300
			(0.435)			(0.397)			(0.891)
			**[0.359]**			**[0.453]**			**[0.702]**
Constant	0.326***	0.376***	0.156*	0.291***	0.326***	0.085**	2.938***	5.310***	0.210
	(0.056)	(0.080)	(0.151)	(0.050)	(0.070)	(0.083)	(0.745)	(1.714)	(0.323)
	**[0.000]**	**[0.000]**	**[0.056]**	**[0.000]**	**[0.000]**	**[0.012]**	**[0.000]**	**[0.000]**	**[0.309]**
**Number of Observations**	1,436	1,436	1,436	1,436	1,436	1,436	371	371	371
**Pseudo R-squared**	0.030	0.062	0.095	0.027	0.056	0.089	0.070	0.131	0.186

Robust clustered standard errors in parentheses. P-values in brackets. Sampling weights were applied. Logistic coefficients reported as odds ratios.

*** p<0.01, ** p<0.05, * p<0.1

Table 2B. LPM Regression Specifications

	(1)	(2)	(3)	(4)	(5)	(6)	(7)	(8)	(9)
	**Household Purchased Latrine**			**Latrine is Installed and Working**			**Latrine has Durable Materials**		
**Specifications:**	(A)	(B)	(C)	(A)	(B)	(C)	(A)	(B)	(C)
**Village Treatment**									
**(SanMark = 0)**									
Choba Only (=1)	-0.056	-0.179***	-0.167***	-0.047	-0.154***	-0.135***	-0.422***	-0.368***	-0.277**
	(0.041)	(0.052)	(0.051)	(0.038)	(0.049)	(0.049)	(0.071)	(0.120)	(0.128)
	**[0.176]**	**[0.001]**	**[0.002]**	**[0.220]**	**[0.002]**	**[0.007]**	**[0.000]**	**[0.003]**	**[0.033]**
Both (=1)	0.140***	0.087	0.091	0.132***	0.093	0.107	-0.229***	-0.255***	-0.191**
	(0.051)	(0.068)	(0.074)	(0.049)	(0.066)	(0.072)	(0.085)	(0.094)	(0.088)
	**[0.007]**	**[0.206]**	**[0.224]**	**[0.008]**	**[0.158]**	**[0.142]**	**[0.008]**	**[0.008]**	**[0.033]**
**Income Groups**									
**(Non-Poor = 0)**									
Near Poor/ID-Poor 3 (=1)		0.109	0.130		0.121	0.142*		-0.137	-0.184*
		(0.080)	(0.079)		(0.075)	(0.074)		(0.094)	(0.097)
		**[0.176]**	**[0.103]**		**[0.110]**	**[0.059]**		**[0.148]**	**[0.060]**
Poor/ID-Poor 1&2 (=1)		-0.149***	-0.165***		-0.128***	-0.142***		-0.473***	-0.441***
		(0.048)	(0.053)		(0.049)	(0.052)		(0.143)	(0.155)
		**[0.002]**	**[0.002]**		**[0.010]**	**[0.008]**		**[0.001]**	**[0.005]**
**Interaction Terms**									
Choba*Near Poor (=1)		0.110	0.087		0.079	0.055		0.083	0.088
		(0.110)	(0.105)		(0.099)	(0.096)		(0.182)	(0.194)
		**[0.319]**	**[0.410]**		**[0.428]**	**[0.568]**		**[0.650]**	**[0.652]**
Choba*Poor (=1)		0.337***	0.326***		0.299***	0.288***		0.078	0.109
		(0.074)	(0.074)		(0.074)	(0.074)		(0.185)	(0.195)
		**[0.000]**	**[0.000]**		**[0.000]**	**[0.000]**		**[0.672]**	**[0.577]**
Both*Near Poor (=1)		-0.041	-0.053		-0.065	-0.079		0.063	0.128
		(0.118)	(0.115)		(0.115)	(0.111)		(0.144)	(0.140)
		**[0.726]**	**[0.644]**		**[0.571]**	**[0.477]**		**[0.660]**	**[0.362]**
Both*Poor (=1)		0.187**	0.185**		0.149*	0.148*		0.286*	0.328*
		(0.082)	(0.084)		(0.080)	(0.082)		(0.170)	(0.177)
		**[0.024]**	**[0.031]**		**[0.067]**	**[0.075]**		**[0.097]**	**[0.067]**
Household Size			0.019***			0.022***			0.000
			(0.007)			(0.007)			(0.017)
			**[0.007]**			**[0.001]**			**[0.982]**
HH Awareness Index			0.065***			0.056***			-0.122***
(Standardized)			(0.014)			(0.014)			(0.028)
			**[0.000]**			**[0.000]**			**[0.000]**
Village Chief Involvement									
Index			-0.001			-0.006			-0.011
(Standardized)			(0.020)			(0.020)			(0.034)
			**[0.948]**			**[0.782]**			**[0.736]**
Village Chief Secondary									
Education or higher (=1)			-0.073*			-0.079**			0.062
			(0.039)			(0.037)			(0.064)
			**[0.063]**			**[0.037]**			**[0.332]**
Village Chief Age			0.002			0.003			0.011**
			(0.003)			(0.003)			(0.004)
			**[0.497]**			**[0.283]**			**[0.020]**
Female Village Chief (=1)			0.063			0.050			0.045
			(0.064)			(0.059)			(0.125)
			**[0.323]**			**[0.394]**			**[0.720]**
Constant	0.246***	0.273***	0.075	0.226***	0.246***	-0.027	0.746***	0.842***	0.166
	(0.032)	(0.043)	(0.176)	(0.030)	(0.040)	(0.167)	(0.048)	(0.044)	(0.312)
	**[0.000]**	**[0.000]**	**[0.670]**	**[0.000]**	**[0.000]**	**[0.873]**	**[0.000]**	**[0.000]**	**[0.597]**
**Number of Observations**	1,436	1,436	1,436	1,436	1,436	1,436	371	371	371
**R-squared**	0.036	0.066	0.101	0.032	0.058	0.092	0.094	0.164	0.226

Robust clustered standard errors in parentheses. P-values in brackets. Sampling weights were applied.
*** p<0.01, ** p<0.05, * p<0.1

Table 3A. Auxiliary Regression Specifications for RA to model HH Purchasing Latrine to account for non-random treatment assignment for Results Reported in Table 5 (Logistic Regression)

	(1)	(2)	(3)	(4)	(5)	(6)	(7)	(8)	(9)
	**HH Purchased Latrine during Project**								
	**Overall**			**Non-Poor**			**Poorest**		
	**SanMark**	**Choba**	**Both**	**SanMark**	**Choba**	**Both**	**SanMark**	**Choba**	**Both**
**<u>Income Group</u>**									
(Non-Poor = 0)									
Near Poor/ID-Poor 3 (=1)	0.610*	1.607***	0.288						
	(0.349)	(0.485)	(0.341)						
	**[0.081]**	**[0.001]**	**[0.398]**						
Poor/ID-Poor 1&2 (=1)	-1.078***	1.266***	0.025						
	(0.361)	(0.394)	(0.289)						
	**[0.003]**	**[0.001]**	**[0.932]**						
Household Size	0.183**	0.037	0.091	0.265***	-0.169	0.134	-0.206	-0.092	0.064
	(0.074)	(0.067)	(0.056)	(0.090)	(0.188)	(0.097)	(0.211)	(0.141)	(0.099)
	**[0.013]**	**[0.579]**	**[0.106]**	**[0.003]**	**[0.368]**	**[0.168]**	**[0.329]**	**[0.512]**	**[0.515]**
HH Awareness Index	0.176	0.448***	0.505***	0.230	0.201	0.334*	0.507*	1.145***	0.918***
(Standardized)	(0.117)	(0.154)	(0.133)	(0.148)	(0.262)	(0.172)	(0.306)	(0.302)	(0.236)
	**[0.133]**	**[0.004]**	**[0.000]**	**[0.122]**	**[0.443]**	**[0.052]**	**[0.098]**	**[0.000]**	**[0.000]**
Village Chief Involvement Index	-0.013	0.173	-0.158	0.178	1.111*	-0.113	-0.771*	-0.424	-0.142
(Standardized)	(0.178)	(0.259)	(0.194)	(0.216)	(0.602)	(0.239)	(0.418)	(0.341)	(0.241)
	**[0.944]**	**[0.504]**	**[0.415]**	**[0.412]**	**[0.065]**	**[0.635]**	**[0.065]**	**[0.214]**	**[0.556]**
Village Chief Secondary									
Education or higher (=1)	-0.723	-0.550	0.063	-0.716	-0.776	0.110	-0.151	-0.715	0.518
	(0.446)	(0.426)	(0.439)	(0.547)	(0.538)	(0.592)	(0.750)	(0.750)	(0.643)
	**[0.105]**	**[0.197]**	**[0.886]**	**[0.191]**	**[0.149]**	**[0.853]**	**[0.840]**	**[0.340]**	**[0.421]**
Village Chief Age	0.022	-0.022	0.015	0.004	-0.075	0.033	0.085	-0.070	0.037
	(0.030)	(0.037)	(0.019)	(0.035)	(0.059)	(0.028)	(0.099)	(0.053)	(0.026)
	**[0.460]**	**[0.552]**	**[0.409]**	**[0.919]**	**[0.208]**	**[0.243]**	**[0.391]**	**[0.185]**	**[0.155]**
Female Village Chief (=1)	0.665	0.344	-0.020	0.748	-6.032***	-0.226	-5.040***	0.440	0.413
	(0.483)	(0.505)	(0.586)	(0.619)	(1.061)	(1.275)	(1.257)	(0.520)	(0.692)
	**[0.169]**	**[0.496]**	**[0.973]**	**[0.227]**	**[0.000]**	**[0.859]**	**[0.000]**	**[0.397]**	**[0.550]**
Constant	-3.246	-1.213	-1.901*	-2.458	2.815	-3.139*	-6.883	3.174	-3.238*
	(1.999)	(2.243)	(1.059)	(2.254)	(3.457)	(1.716)	(6.963)	(3.089)	(1.713)
	**[0.104]**	**[0.589]**	**[0.073]**	**[0.275]**	**[0.416]**	**[0.067]**	**[0.323]**	**[0.304]**	**[0.059]**
**Number of Observations**	1,436	1,436	1,436	760	760	760	401	401	401

Robust clustered standard errors in parentheses. P-values in brackets. Sampling weights were applied. Coefficients reported as logistic coefficients.
*** p<0.01, ** p<0.05, * p<0.1

Table 3A. (continued) Auxiliary Regression Specifications for RA to model HH Installation of Latrine to account for non-random treatment assignment for Results Reported in Table 5 (Logistic Regression)

	(1)	(2)	(3)	(4)	(5)	(6)	(7)	(8)	(9)
	**HH Purchased Latrine during Project**								
	**Overall**			**Non-Poor**			**Poorest**		
	**SanMark**	**Choba**	**Both**	**SanMark**	**Choba**	**Both**	**SanMark**	**Choba**	**Both**
**<u>Income Group</u>**									
(Non-Poor = 0)									
Near Poor/ID-Poor 3 (=1)	0.684**	1.542***	0.227						
	(0.336)	(0.468)	(0.345)						
	**[0.042]**	**[0.001]**	**[0.511]**						
Poor/ID-Poor 1&2 (=1)	-0.978**	1.213***	-0.042						
	(0.393)	(0.403)	(0.285)						
	**[0.013]**	**[0.003]**	**[0.883]**						
Household Size	0.197**	0.053	0.127**	0.279***	-0.118	0.190**	-0.194	-0.123	0.131
	(0.077)	(0.067)	(0.056)	(0.093)	(0.185)	(0.095)	(0.217)	(0.135)	(0.110)
	**[0.011]**	**[0.428]**	**[0.024]**	**[0.003]**	**[0.523]**	**[0.045]**	**[0.370]**	**[0.363]**	**[0.237]**
HH Awareness Index	0.125	0.405***	0.497***	0.162	0.166	0.320*	0.614*	1.102***	0.930***
(Standardized)	(0.115)	(0.153)	(0.134)	(0.135)	(0.265)	(0.177)	(0.317)	(0.325)	(0.269)
	**[0.277]**	**[0.008]**	**[0.000]**	**[0.231]**	**[0.532]**	**[0.071]**	**[0.053]**	**[0.001]**	**[0.001]**
Village Chief Involvement Index	-0.035	0.223	-0.217	0.150	1.334**	-0.205	-0.825*	-0.430	-0.121
(Standardized)	(0.175)	(0.260)	(0.197)	(0.212)	(0.596)	(0.247)	(0.444)	(0.349)	(0.256)
	**[0.841]**	**[0.392]**	**[0.271]**	**[0.479]**	**[0.025]**	**[0.407]**	**[0.063]**	**[0.218]**	**[0.638]**
Village Chief Secondary									
Education or higher (=1)	-0.757*	-0.470	-0.071	-0.789	-0.706	-0.011	-0.149	-0.546	0.068
	(0.447)	(0.424)	(0.452)	(0.528)	(0.514)	(0.624)	(0.756)	(0.750)	(0.671)
	**[0.090]**	**[0.268]**	**[0.875]**	**[0.136]**	**[0.170]**	**[0.986]**	**[0.844]**	**[0.467]**	**[0.919]**
Village Chief Age	0.028	-0.037	0.024	0.014	-0.073	0.046	0.098	-0.092*	0.038
	(0.031)	(0.035)	(0.019)	(0.033)	(0.060)	(0.029)	(0.100)	(0.055)	(0.029)
	**[0.374]**	**[0.298]**	**[0.211]**	**[0.660]**	**[0.222]**	**[0.110]**	**[0.327]**	**[0.098]**	**[0.198]**
Female Village Chief (=1)	0.733	0.110	-0.013	0.857	-5.499***	-0.019	-5.029***	0.552	-0.108
	(0.533)	(0.445)	(0.598)	(0.657)	(0.970)	(1.229)	(1.297)	(0.567)	(0.424)
	**[0.169]**	**[0.805]**	**[0.983]**	**[0.192]**	**[0.000]**	**[0.987]**	**[0.000]**	**[0.330]**	**[0.799]**
Constant	-3.833*	-0.452	-2.655**	-3.371	2.299	-4.235**	-7.894	4.410	-3.745*
	(2.050)	(2.118)	(1.131)	(2.118)	(3.414)	(1.686)	(7.071)	(3.117)	(2.006)
	**[0.062]**	**[0.831]**	**[0.019]**	**[0.111]**	**[0.501]**	**[0.012]**	**[0.264]**	**[0.157]**	**[0.062]**
**Number of Observations**	1,436	1,436	1,436	760	760	760	401	401	401

Robust clustered standard errors in parentheses. P-values in brackets. Sampling weights were applied. Coefficients reported as logistic coefficients.
*** p<0.01, ** p<0.05, * p<0.1

Table 3B. Auxiliary Regression Specifications for RA to model HH Latrine Purchases to account for non-random treatment assignment for Results Reported in Table 5 (LPM)

	(1)	(2)	(3)	(4)	(5)	(6)	(7)	(8)	(9)
	**HH Purchased Latrine during Project**								
	**Overall**			**Non-Poor**			**Poorest**		
	**SanMark**	**Choba**	**Both**	**SanMark**	**Choba**	**Both**	**SanMark**	**Choba**	**Both**
**Income Group**									
(Non-Poor = 0)									
Near Poor/ID-Poor 3 (=1)	0.124*	0.223***	0.065						
	(0.075)	(0.069)	(0.077)						
	**[0.097]**	**[0.001]**	**[0.394]**						
Poor/ID-Poor 1&2 (=1)	-0.161***	0.168***	0.005						
	(0.051)	(0.053)	(0.065)						
	**[0.002]**	**[0.001]**	**[0.936]**						
Household Size	0.032***	0.005	0.020	0.051***	-0.012	0.029	-0.019	-0.016	0.012
	(0.012)	(0.009)	(0.012)	(0.017)	(0.012)	(0.021)	(0.021)	(0.025)	(0.021)
	**[0.010]**	**[0.572]**	**[0.107]**	**[0.002]**	**[0.322]**	**[0.166]**	**[0.347]**	**[0.513]**	**[0.550]**
HH Awareness Index	0.030	0.060***	0.113***	0.043	0.011	0.073*	0.041	0.169***	0.191***
(Standardized)	(0.021)	(0.022)	(0.028)	(0.027)	(0.021)	(0.038)	(0.027)	(0.039)	(0.040)
	**[0.155]**	**[0.006]**	**[0.000]**	**[0.120]**	**[0.611]**	**[0.055]**	**[0.131]**	**[0.000]**	**[0.000]**
Village Chief Involvement Index	-0.003	0.021	-0.033	0.034	0.080**	-0.022	-0.059*	-0.065	-0.028
(Standardized)	(0.030)	(0.034)	(0.044)	(0.039)	(0.036)	(0.054)	(0.033)	(0.060)	(0.048)
	**[0.923]**	**[0.525]**	**[0.449]**	**[0.394]**	**[0.026]**	**[0.675]**	**[0.069]**	**[0.274]**	**[0.553]**
Village Chief Secondary									
Education or higher (=1)	-0.120*	-0.054	0.019	-0.125	-0.043	0.033	-0.012	-0.104	0.119
	(0.064)	(0.044)	(0.098)	(0.089)	(0.038)	(0.133)	(0.093)	(0.099)	(0.132)
	**[0.062]**	**[0.213]**	**[0.848]**	**[0.161]**	**[0.255]**	**[0.806]**	**[0.898]**	**[0.296]**	**[0.369]**
Village Chief Age	0.004	-0.002	0.003	0.001	-0.007	0.007	0.006	-0.012	0.008
	(0.005)	(0.005)	(0.004)	(0.007)	(0.005)	(0.006)	(0.008)	(0.009)	(0.005)
	**[0.430]**	**[0.668]**	**[0.410]**	**[0.922]**	**[0.193]**	**[0.258]**	**[0.433]**	**[0.180]**	**[0.127]**
Female Village Chief (=1)	0.126	0.074	-0.003	0.159	-0.143**	-0.040	0.036	0.077	0.077
	(0.097)	(0.088)	(0.128)	(0.136)	(0.060)	(0.248)	(0.106)	(0.106)	(0.146)
	**[0.196]**	**[0.399]**	**[0.982]**	**[0.240]**	**[0.016]**	**[0.871]**	**[0.733]**	**[0.469]**	**[0.596]**
Constant	-0.123	0.207	0.075	0.002	0.593*	-0.189	-0.174	1.032*	-0.195
	(0.333)	(0.322)	(0.233)	(0.428)	(0.316)	(0.376)	(0.497)	(0.546)	(0.345)
	**[0.712]**	**[0.520]**	**[0.748]**	**[0.997]**	**[0.060]**	**[0.615]**	**[0.727]**	**[0.059]**	**[0.572]**
**Number of Observations**	1,436	1,436	1,436	760	760	760	401	401	401

Robust clustered standard errors in parentheses. P-values in brackets. Sampling weights were applied.
*** p<0.01, ** p<0.05, * p<0.1

Table 3B. (continued) Auxiliary Regression Specifications for RA to account for HH Installation of Latrine for non-random treatment assignment for Results Reported in Table 5 (LPM)

	(1)	(2)	(3)	(4)	(5)	(6)	(7)	(8)	(9)
	**HH Purchased Latrine during Project**								
		**Overall**			**Non-Poor**			**Poorest**	
	**SanMark**	**Choba**	**Both**	**SanMark**	**Choba**	**Both**	**SanMark**	**Choba**	**Both**
<u>**Income Group**</u>									
(Non-Poor = 0)									
Near Poor/ID-Poor 3 (=1)	0.134*	0.206***	0.051						
	(0.070)	(0.063)	(0.075)						
	**[0.055]**	**[0.001]**	**[0.500]**						
Poor/ID-Poor 1&2 (=1)	-0.138***	0.155***	-0.009						
	(0.051)	(0.053)	(0.061)						
	**[0.007]**	**[0.003]**	**[0.879]**						
Household Size	0.032***	0.007	0.027**	0.050***	-0.008	0.039**	-0.017	-0.022	0.024
	(0.012)	(0.009)	(0.012)	(0.016)	(0.012)	(0.019)	(0.021)	(0.023)	(0.022)
	**[0.007]**	**[0.434]**	**[0.022]**	**[0.002]**	**[0.467]**	**[0.042]**	**[0.403]**	**[0.347]**	**[0.264]**
HH Awareness Index	0.020	0.052**	0.105***	0.029	0.008	0.065*	0.047*	0.153***	0.180***
(Standardized)	(0.020)	(0.020)	(0.028)	(0.023)	(0.020)	(0.037)	(0.026)	(0.039)	(0.042)
	**[0.297]**	**[0.010]**	**[0.000]**	**[0.218]**	**[0.712]**	**[0.079]**	**[0.075]**	**[0.000]**	**[0.000]**
Village Chief Involvement Index	-0.006	0.028	-0.043	0.027	0.088***	-0.038	-0.058*	-0.063	-0.018
(Standardized)	(0.028)	(0.033)	(0.044)	(0.036)	(0.033)	(0.054)	(0.031)	(0.060)	(0.048)
	**[0.834]**	**[0.392]**	**[0.331]**	**[0.461]**	**[0.009]**	**[0.479]**	**[0.061]**	**[0.291]**	**[0.716]**
Village Chief Secondary									
Education or higher (=1)	-0.117**	-0.045	-0.004	-0.125	-0.037	0.019	-0.007	-0.077	0.030
	(0.059)	(0.042)	(0.097)	(0.078)	(0.036)	(0.132)	(0.092)	(0.098)	(0.131)
	**[0.049]**	**[0.289]**	**[0.969]**	**[0.107]**	**[0.309]**	**[0.883]**	**[0.937]**	**[0.429]**	**[0.821]**
Village Chief Age	0.005	-0.004	0.005	0.003	-0.007	0.009	0.007	-0.015*	0.008
	(0.005)	(0.005)	(0.004)	(0.006)	(0.005)	(0.006)	(0.007)	(0.009)	(0.006)
	**[0.355]**	**[0.367]**	**[0.218]**	**[0.670]**	**[0.191]**	**[0.133]**	**[0.384]**	**[0.080]**	**[0.162]**
Female Village Chief (=1)	0.131	0.032	0.001	0.175	-0.134**	0.003	0.056	0.098	-0.034
	(0.107)	(0.070)	(0.125)	(0.143)	(0.057)	(0.235)	(0.106)	(0.112)	(0.077)
	**[0.222]**	**[0.652]**	**[0.996]**	**[0.221]**	**[0.018]**	**[0.989]**	**[0.600]**	**[0.382]**	**[0.657]**
Constant	-0.197	0.317	-0.067	-0.144	0.572*	-0.374	-0.231	1.220**	-0.257
	(0.323)	(0.295)	(0.235)	(0.375)	(0.314)	(0.353)	(0.496)	(0.521)	(0.366)
	**[0.541]**	**[0.282]**	**[0.777]**	**[0.700]**	**[0.068]**	**[0.290]**	**[0.641]**	**[0.019]**	**[0.483]**
**Number of Observations**	1,436	1,436	1,436	760	760	760	401	401	401

Robust clustered standard errors in parentheses. P-values in brackets. Sampling weights were applied.
*** p<0.01, ** p<0.05, * p<0.1

Table 3C. Auxiliary Regression Specifications for IPW & IPWRA to account for non-random treatment assignment for Results Reported in Table 5 (Multinomial Logistic Regression)

	(1)	(2)	(3)	(4)	(5)	(6)
	**Overall**		**Non-Poor**		**Poor**	
	**Choba**	**Both**	**Choba**	**Both**	**Choba**	**Both**
**Income Groups**						
**(Non-Poor = 0)**						
Near Poor/ID-Poor 3 (=1)	0.885***	0.997***				
	(0.224)	(0.273)				
	**[0.000]**	**[0.000]**				
Poor/ID-Poor 1&2 (=1)	-0.072	0.180				
	(0.169)	(0.177)				
	**[0.672]**	**[0.308]**				
Household Size	-0.056	-0.073	-0.116**	-0.084	-0.069	-0.083
	(0.043)	(0.048)	(0.056)	(0.062)	(0.086)	(0.088)
	**[0.195]**	**[0.127]**	**[0.040]**	**[0.177]**	**[0.422]**	**[0.350]**
HH Awareness Index	-0.040	-0.001	-0.063	-0.039	-0.007	-0.063
(Standardized)	(0.147)	(0.162)	(0.189)	(0.208)	(0.180)	(0.190)
	**[0.784]**	**[0.997]**	**[0.738]**	**[0.850]**	**[0.969]**	**[0.740]**
Village Chief Involvement Index	0.829***	1.196***	0.887***	1.285***	0.699**	1.263***
(Standardized)	(0.298)	(0.341)	(0.305)	(0.362)	(0.328)	(0.355)
	**[0.005]**	**[0.000]**	**[0.004]**	**[0.000]**	**[0.033]**	**[0.000]**
Village Chief Secondary Education or higher (=1)	0.260	-0.650	0.283	-0.641	0.183	-0.566
	(0.706)	(0.722)	(0.767)	(0.791)	(0.684)	(0.694)
	**[0.713]**	**[0.367]**	**[0.712]**	**[0.418]**	**[0.789]**	**[0.415]**
Village Chief Age	-0.154***	-0.209***	-0.155**	-0.204***	-0.138**	-0.189**
	(0.059)	(0.066)	(0.062)	(0.070)	(0.070)	(0.076)
	**[0.009]**	**[0.001]**	**[0.013]**	**[0.004]**	**[0.048]**	**[0.013]**
Female Village Chief (=1)	-0.537	-2.002*	-0.972	-1.948	-0.079	-1.586
	(1.131)	(1.166)	(1.148)	(1.252)	(1.085)	(1.309)
	**[0.635]**	**[0.086]**	**[0.397]**	**[0.120]**	**[0.942]**	**[0.226]**
Constant	9.837***	13.208***	10.270***	12.969***	8.812*	12.184**
	(3.702)	(4.035)	(3.876)	(4.269)	(4.528)	(4.793)
	[0.008]	[0.001]	[0.008]	[0.002]	[0.052]	[0.011]
**Number of Observations**	1,436	1,436	760	760	401	401

Robust clustered standard errors in parentheses. P-values in brackets. Sampling weights were applied. Coefficients reported as logistic coefficients. Reference category for multinomial logistic regression above is SanMark only villages.
*** p<0.01, ** p<0.05, * p<0.1

Table 3D. Auxiliary Regression Specifications for IPWRA to model HH Latrine Purchases to account for non-random treatment assignment for non-random treatment assignment for Results Reported in Table 5 (Logistic Regression)

	(1)	(2)	(3)	(4)	(5)	(6)	(7)	(8)	(9)
					**Household Purchased Latrine**				
**VARIABLES**		**overall**			**non-poor**			**poorest**	
<u>**Income Groups**</u>									
**(Non-Poor = 0)**									
Near Poor/ID-Poor 3 (=1)	0.565	1.607***	0.165						
	(0.369)	(0.485)	(0.408)						
	**[0.126]**	**[0.001]**	**[0.686]**						
Poor/ID-Poor 1&2 (=1)	-2.516***	1.266***	-0.118						
	(0.852)	(0.394)	(0.380)						
	**[0.003]**	**[0.001]**	**[0.756]**						
Household Size	0.697*	0.037	0.065	0.767**	-0.169	0.094	-0.413	-0.092	-0.007
	(0.361)	(0.067)	(0.050)	(0.378)	(0.188)	(0.116)	(0.272)	(0.141)	(0.160)
	**[0.054]**	**[0.579]**	**[0.197]**	**[0.043]**	**[0.368]**	**[0.416]**	**[0.129]**	**[0.512]**	**[0.967]**
HH Awareness Index	0.016	0.448***	0.574***	0.134	0.201	0.311	0.951***	1.145***	1.091***
(Standardized)	(0.197)	(0.154)	(0.145)	(0.243)	(0.262)	(0.203)	(0.331)	(0.302)	(0.234)
	**[0.934]**	**[0.004]**	**[0.000]**	**[0.581]**	**[0.443]**	**[0.126]**	**[0.004]**	**[0.000]**	**[0.000]**
Village Chief Involvement Index	-0.207	0.173	-0.248	-0.015	1.111*	-0.182	-0.959***	-0.424	-0.096
(Standardized)	(0.200)	(0.259)	(0.187)	(0.260)	(0.602)	(0.239)	(0.306)	(0.341)	(0.217)
	**[0.300]**	**[0.504]**	**[0.184]**	**[0.954]**	**[0.065]**	**[0.447]**	**[0.002]**	**[0.214]**	**[0.659]**
Village Chief Secondary Education or higher (=1)	-1.690***	-0.550	0.076	-1.432**	-0.776	0.230	-1.481	-0.715	0.263
	(0.527)	(0.426)	(0.537)	(0.729)	(0.538)	(0.724)	(1.153)	(0.750)	(0.595)
	**[0.001]**	**[0.197]**	**[0.887]**	**[0.050]**	**[0.149]**	**[0.751]**	**[0.199]**	**[0.340]**	**[0.659]**
Village Chief Age	0.074*	-0.022	0.045*	0.087	-0.075	0.075**	-0.063	-0.070	0.046*
	(0.043)	(0.037)	(0.023)	(0.061)	(0.059)	(0.032)	(0.188)	(0.053)	(0.025)
	**[0.088]**	**[0.552]**	**[0.056]**	**[0.151]**	**[0.208]**	**[0.020]**	**[0.737]**	**[0.185]**	**[0.065]**
Female Village Chief (=1)	0.809	0.344	0.788	1.127	-5.531***	1.501	-8.725*	0.440	-0.184
	(0.814)	(0.505)	(0.487)	(1.209)	(1.043)	(1.060)	(4.882)	(0.520)	(0.668)
	**[0.320]**	**[0.496]**	**[0.106]**	**[0.351]**	**[0.000]**	**[0.157]**	**[0.074]**	**[0.397]**	**[0.783]**
Constant	-8.994**	-1.213	-3.477**	-10.251*	2.814	-5.586***	3.144	3.174	-3.413*
	(4.444)	(2.243)	(1.391)	(5.451)	(3.456)	(2.085)	(12.244)	(3.089)	(1.879)
	**[0.043]**	**[0.589]**	**[0.012]**	**[0.060]**	**[0.416]**	**[0.007]**	**[0.797]**	**[0.304]**	**[0.069]**
**Number of Observations**	1,436	1,436	1,436	760	760	760	401	401	401

Robust clustered standard errors in parentheses. P-values in brackets. Sampling weights were applied. Coefficients reported as logistic coefficients. For specifications to account for non-random treatment assignment (IPW portion of IPWRA doubly robust method), refer to Annex Table 3(C).
*** $p<0.01$, ** $p<0.05$, * $p<0.1$

Table 3D. (continued) Auxiliary Regression Specifications for IPWRA to model HH Latrine Installation to account for non-random treatment assignment for non-random treatment assignment for Results Reported in Table 5 (Logistic Regression)

	(1)	(2)	(3)	(4)	(5)	(6)	(7)	(8)	(9)
					**Latrine is Installed and Working**				
**VARIABLES**		**overall**			**non-poor**			**poorest**	
**<u>Income Groups</u>**									
**(Non-Poor = 0)**									
Near Poor/ID-Poor 3 (=1)	0.780*	1.542***	0.127						
	(0.438)	(0.468)	(0.418)						
	**[0.075]**	**[0.001]**	**[0.762]**						
Poor/ID-Poor 1&2 (=1)	-2.377***	1.213***	-0.269						
	(0.888)	(0.403)	(0.380)						
	**[0.007]**	**[0.003]**	**[0.480]**						
Household Size	0.717*	0.053	0.086	0.787**	-0.118	0.114	-0.400	-0.123	0.061
	(0.372)	(0.067)	(0.054)	(0.395)	(0.185)	(0.117)	(0.275)	(0.135)	(0.179)
	**[0.054]**	**[0.428]**	**[0.113]**	**[0.046]**	**[0.523]**	**[0.330]**	**[0.146]**	**[0.363]**	**[0.736]**
HH Awareness Index	-0.047	0.405***	0.583***	0.000	0.166	0.311	1.146***	1.102***	1.195***
(Standardized)	(0.203)	(0.153)	(0.146)	(0.221)	(0.265)	(0.206)	(0.315)	(0.325)	(0.269)
	**[0.815]**	**[0.008]**	**[0.000]**	**[0.999]**	**[0.532]**	**[0.131]**	**[0.000]**	**[0.001]**	**[0.000]**
Village Chief Involvement Index	-0.205	0.223	-0.324*	-0.041	1.334**	-0.264	-0.986***	-0.430	-0.213
(Standardized)	(0.174)	(0.260)	(0.192)	(0.242)	(0.597)	(0.242)	(0.319)	(0.349)	(0.223)
	**[0.239]**	**[0.392]**	**[0.092]**	**[0.865]**	**[0.025]**	**[0.276]**	**[0.002]**	**[0.218]**	**[0.340]**
Village Chief Secondary Education or higher (=1)	-1.770***	-0.470	-0.020	-1.408*	-0.706	0.187	-1.455	-0.546	-0.289
	(0.621)	(0.424)	(0.543)	(0.768)	(0.514)	(0.741)	(1.158)	(0.750)	(0.609)
	**[0.004]**	**[0.268]**	**[0.970]**	**[0.067]**	**[0.170]**	**[0.800]**	**[0.209]**	**[0.467]**	**[0.635]**
Village Chief Age	0.069	-0.037	0.057**	0.089	-0.073	0.086***	-0.052	-0.092*	0.085**
	(0.043)	(0.035)	(0.024)	(0.055)	(0.060)	(0.033)	(0.195)	(0.055)	(0.036)
	**[0.106]**	**[0.298]**	**[0.016]**	**[0.104]**	**[0.222]**	**[0.008]**	**[0.791]**	**[0.098]**	**[0.017]**
Female Village Chief (=1)	0.789	0.110	0.857*	1.211	-8.885***	1.668	-8.510*	0.552	-0.239
	(0.843)	(0.445)	(0.517)	(1.089)	(0.955)	(1.036)	(5.064)	(0.567)	(0.557)
	**[0.349]**	**[0.805]**	**[0.097]**	**[0.266]**	**[0.000]**	**[0.107]**	**[0.093]**	**[0.330]**	**[0.669]**
Constant	-9.033**	-0.452	-4.374***	-10.736**	2.299	-6.403***	2.164	4.410	-6.216**
	(4.374)	(2.118)	(1.429)	(5.175)	(3.417)	(2.053)	(12.659)	(3.117)	(2.601)
	**[0.039]**	**[0.831]**	**[0.002]**	**[0.038]**	**[0.501]**	**[0.002]**	**[0.864]**	**[0.157]**	**[0.017]**
**Number of Observations**	1,436	1,436	1,436	760	760	760	401	401	401

Robust clustered standard errors in parentheses. P-values in brackets. Sampling weights were applied. Coefficients reported as logistic coefficients. For specifications to account for non-random treatment assignment (IPW portion of IPWRA doubly robust method), refer to Annex Table 3(C).
*** p<0.01, ** p<0.05, * p<0.1

Table 3E. Auxiliary Regression Specifications for IPWRA to model HH Latrine Purchases to account for non-random treatment assignment for non-random treatment assignment for Results Reported in Table 5 (LPM)

	(1)	(2)	(3)	(4)	(5)	(6)	(7)	(8)	(9)
					Household Purchased Latrine				
**VARIABLES**		**overall**			**non-poor**			**poorest**	
**<u>Income Groups</u>**									
**(Non-Poor = 0)**									
Near Poor/ID-Poor 3 (=1)	0.080	0.223***	0.036						
	(0.059)	(0.069)	(0.090)						
	**[0.173]**	**[0.001]**	**[0.689]**						
Poor/ID-Poor 1&2 (=1)	-0.212***	0.168***	-0.028						
	(0.044)	(0.053)	(0.083)						
	**[0.000]**	**[0.001]**	**[0.739]**						
Household Size	0.084***	0.005	0.014	0.096***	-0.012	0.021	-0.013	-0.016	-0.001
	(0.022)	(0.009)	(0.011)	(0.022)	(0.012)	(0.024)	(0.013)	(0.025)	(0.031)
	**[0.000]**	**[0.572]**	**[0.204]**	**[0.000]**	**[0.322]**	**[0.382]**	**[0.306]**	**[0.513]**	**[0.978]**
HH Awareness Index	-0.019	0.060***	0.127***	0.010	0.011	0.065	0.022	0.169***	0.224***
(Standardized)	(0.042)	(0.022)	(0.031)	(0.045)	(0.021)	(0.044)	(0.020)	(0.039)	(0.039)
	**[0.654]**	**[0.006]**	**[0.000]**	**[0.832]**	**[0.611]**	**[0.141]**	**[0.268]**	**[0.000]**	**[0.000]**
Village Chief Involvement Index	-0.028	0.021	-0.051	0.007	0.080**	-0.033	-0.072***	-0.065	-0.019
(Standardized)	(0.025)	(0.034)	(0.041)	(0.038)	(0.036)	(0.049)	(0.025)	(0.060)	(0.041)
	**[0.256]**	**[0.525]**	**[0.219]**	**[0.860]**	**[0.026]**	**[0.509]**	**[0.005]**	**[0.274]**	**[0.637]**
Village Chief Secondary Education or higher (=1)	-0.216***	-0.054	0.030	-0.169**	-0.043	0.075	-0.104*	-0.104	0.058
	(0.044)	(0.044)	(0.118)	(0.081)	(0.038)	(0.155)	(0.054)	(0.099)	(0.120)
	**[0.000]**	**[0.213]**	**[0.798]**	**[0.037]**	**[0.255]**	**[0.628]**	**[0.052]**	**[0.296]**	**[0.630]**
Village Chief Age	0.008**	-0.002	0.009*	0.010	-0.007	0.016**	-0.000	-0.012	0.010**
	(0.004)	(0.005)	(0.005)	(0.006)	(0.005)	(0.006)	(0.002)	(0.009)	(0.005)
	**[0.047]**	**[0.668]**	**[0.055]**	**[0.122]**	**[0.193]**	**[0.013]**	**[0.921]**	**[0.180]**	**[0.039]**
Female Village Chief (=1)	0.092	0.074	0.179	0.158	-0.143**	0.330	-0.089	0.077	-0.053
	(0.102)	(0.088)	(0.112)	(0.162)	(0.060)	(0.224)	(0.068)	(0.106)	(0.131)
	**[0.367]**	**[0.399]**	**[0.108]**	**[0.327]**	**[0.016]**	**[0.141]**	**[0.190]**	**[0.469]**	**[0.685]**
Constant	-0.588**	0.207	-0.244	-0.789*	0.593*	-0.674*	0.197	1.032*	-0.225
	(0.286)	(0.322)	(0.291)	(0.431)	(0.316)	(0.400)	(0.161)	(0.546)	(0.361)
	**[0.040]**	**[0.520]**	**[0.401]**	**[0.067]**	**[0.060]**	**[0.092]**	**[0.222]**	**[0.059]**	**[0.533]**
**Number of Observations**	1,436	1,436	1,436	760	760	760	401	401	401

Robust clustered standard errors in parentheses. P-values in brackets. Sampling weights were applied. For specifications to account for non-random treatment assignment (IPW portion of IPWRA doubly robust method), refer to Annex Table 3(C).
*** p<0.01, ** p<0.05, * p<0.1

Table 3E. (continued) Auxiliary Regression Specifications for IPWRA to adjust HH Latrine Installation for non-random treatment assignment for non-random treatment assignment for Results Reported in Table 5 (LPM)

	(1)	(2)	(3)	(4)	(5)	(6)	(7)	(8)	(9)
					**Household Purchased Latrine**				
**VARIABLES**		**overall**			**non-poor**			**poorest**	
**Income Groups**									
**(Non-Poor = 0)**									
Near Poor/ID-Poor 3 (=1)	0.106*	0.206***	0.027						
	(0.062)	(0.063)	(0.090)						
	**[0.086]**	**[0.001]**	**[0.768]**						
Poor/ID-Poor 1&2 (=1)	-0.191***	0.155***	-0.059						
	(0.037)	(0.053)	(0.080)						
	**[0.000]**	**[0.003]**	**[0.459]**						
Household Size	0.084***	0.007	0.018	0.094***	-0.008	0.025	-0.013	-0.022	0.011
	(0.023)	(0.009)	(0.011)	(0.023)	(0.012)	(0.023)	(0.013)	(0.023)	(0.032)
	**[0.000]**	**[0.434]**	**[0.117]**	**[0.000]**	**[0.467]**	**[0.290]**	**[0.308]**	**[0.347]**	**[0.728]**
HH Awareness Index	-0.027	0.052**	0.123***	-0.011	0.008	0.061	0.023	0.153***	0.224***
(Standardized)	(0.040)	(0.020)	(0.030)	(0.039)	(0.020)	(0.044)	(0.021)	(0.039)	(0.040)
	**[0.500]**	**[0.010]**	**[0.000]**	**[0.778]**	**[0.712]**	**[0.159]**	**[0.258]**	**[0.000]**	**[0.000]**
Village Chief Involvement Index	-0.026	0.028	-0.062	0.002	0.088***	-0.045	-0.066***	-0.063	-0.031
(Standardized)	(0.021)	(0.033)	(0.041)	(0.033)	(0.033)	(0.048)	(0.025)	(0.060)	(0.038)
	**[0.221]**	**[0.392]**	**[0.133]**	**[0.947]**	**[0.009]**	**[0.345]**	**[0.009]**	**[0.291]**	**[0.421]**
Village Chief Secondary Education or higher (=1)	-0.209***	-0.045	0.019	-0.145**	-0.037	0.075	-0.095*	-0.077	-0.033
	(0.045)	(0.042)	(0.117)	(0.070)	(0.036)	(0.154)	(0.054)	(0.098)	(0.117)
	**[0.000]**	**[0.289]**	**[0.874]**	**[0.040]**	**[0.309]**	**[0.625]**	**[0.077]**	**[0.429]**	**[0.779]**
Village Chief Age	0.007	-0.004	0.011**	0.010	-0.007	0.017***	-0.000	-0.015*	0.016***
	(0.004)	(0.005)	(0.005)	(0.006)	(0.005)	(0.006)	(0.002)	(0.009)	(0.006)
	**[0.125]**	**[0.367]**	**[0.017]**	**[0.106]**	**[0.191]**	**[0.005]**	**[0.990]**	**[0.080]**	**[0.006]**
Female Village Chief (=1)	0.082	0.032	0.189	0.165	-0.134**	0.354*	-0.079	0.098	-0.068
	(0.114)	(0.070)	(0.117)	(0.153)	(0.057)	(0.213)	(0.070)	(0.112)	(0.096)
	**[0.475]**	**[0.652]**	**[0.108]**	**[0.283]**	**[0.018]**	**[0.096]**	**[0.258]**	**[0.382]**	**[0.481]**
Constant	-0.550*	0.317	-0.386	-0.797**	0.572*	-0.782**	0.176	1.220**	-0.644
	(0.295)	(0.295)	(0.284)	(0.384)	(0.314)	(0.374)	(0.165)	(0.521)	(0.424)
	**[0.062]**	**[0.282]**	**[0.174]**	**[0.038]**	**[0.068]**	**[0.036]**	**[0.286]**	**[0.019]**	**[0.129]**
**Number of Observations**	1,436	1,436	1,436	760	760	760	401	401	401

Robust clustered standard errors in parentheses. P-values in brackets. Sampling weights were applied. For specifications to account for non-random treatment assignment (IPW portion of IPWRA doubly robust method), refer to Annex Table 3(C)

*** p<0.01, ** p<0.05, * p<0.1
