# Supplementary material for: Strategic complements: Poverty-targeted subsidy programs show additive benefits on household toilet purchases in rural Cambodia when coupled with sanitation marketing
Source: PLoS One. 2022 Jun 15;17(6):e0269980. doi: 10.1371/journal.pone.0269980 (PMC9200298; doi:10.1371/journal.pone.0269980)
Supplement: S2 Table — (PDF) [file pone.0269980.s003.pdf]

S2 Table. Near-poor Household responses when they were asked if they think that they will be offered a subsidy by another organization in the future.

Table A. Near-poor Household's responses when they were asked if they think that they will be offered a subsidy by another organization in the future.

(1)<br>Do households think that another organization will offer a subsidy in the community in the next year?	(2)<br>Frequency	(3)<br>Percentage
**Yes**	20	7 %
**No**	10	4 %
**Don't Know**	245	89 %
**Total**	275	100%

Among all near poor-households included in the analysis (N = 275 of 1,436 households)
